# Supplementary material for: Mapping cumulative impacts to coastal ecosystem services in British Columbia
Source: PLoS One. 2020 May 4;15(5):e0220092. doi: 10.1371/journal.pone.0220092 (PMC7197858; doi:10.1371/journal.pone.0220092)
Supplement: S1 Table — (DOCX) [file pone.0220092.s001.docx]

S1 Table. Erosion risk to different coastal classes

| **Shorezone Erosion Risk (1 is low, 5 high)** | |
| --- | --- |
| **Rank** | **COASTAL_CLASS_NAME** |
| 3 | Undefined |
| 1 | Rock Ramp, wide > 30m |
| 1 | Rock Platform, wide > 30m |
| 1 | Rock Cliff, narrow < 30m |
| 1 | Rock Ramp, narrow < 30m |
| 1 | Rock Platform, narrow < 30m |
| 4 | Rock Ramp with Gravel Beach, wide > 30m |
| 4 | Rock Platform with Gravel Beach, wide > 30m |
| 3 | Rock Cliff with Gravel Beach, narrow < 30m |
| 4 | Rock Ramp with Gravel Beach, narrow < 30m |
| 4 | Rock Platform with Gravel Beach, narrow < 30m |
| 5 | Rock Ramp with Sand and Gravel Beach, wide > 30m |
| 5 | Rock Platform with Sand and Gravel Beach, wide > 30m |
| 4 | Rock Cliff with Sand and Gravel Beach, narrow < 30m |
| 5 | Rock Ramp with Sand and Gravel Beach, narrow < 30m |
| 4 | Rock Platform with Sand and Gravel Beach, narrow < 30m |
| 5 | Rock Ramp with Sand Beach, wide > 30m |
| 5 | Rock Platform with Sand Beach, wide > 30m |
| 5 | Rock Platform with Sand Beach, wide > 30m |
| 5 | Rock Ramp with Sand Beach, narrow < 30m |
| 5 | Rock Platform with sand Beach, narrow < 30m |
| 4 | Gravel Flat, wide > 30m |
| 4 | Gravel Beach, narrow < 30m |
| 4 | Gravel Flat or Fan, narrow < 30m |
| 5 | Sand and Gravel Flat or Fan, wide > 30m |
| 5 | Sand and Gravel Beach, narrow < 30m |
| 5 | Sand and Gravel Flat or Fan, narrow < 30m |
| 5 | Sand Beach, wide > 30m |
| 5 | Sand Flat, wide > 30m |
| 5 | Mud Flat, wide > 30m |
| 5 | Sand Beach, narrow < 30m |
| 4 | Estuary (Organics/Fines) |
| 2 | Man made, permeable |
| 1 | Man made impermeable |
| 4 | Channel |
| 4 | Hanging Lagoon |
