# Supplementary material for: Mapping cumulative impacts to coastal ecosystem services in British Columbia
Source: PLoS One. 2020 May 4;15(5):e0220092. doi: 10.1371/journal.pone.0220092 (PMC7197858; doi:10.1371/journal.pone.0220092)
Supplement: S2 Table — (DOCX) [file pone.0220092.s002.docx]

S2 Table . Data files, sources, and resolution used to map impacts and ecosystem services

| Ecosystem Service | Data | Description | Resolution |
| --- | --- | --- | --- |
| Coastal Aesthetics | Campsites_coastal | coastal British Columbia campsites | 1: 40,000 |
|  | Coastal_parks_WGS | Parks in Coastal British Columbia | not listed |
|  | Population_raster | global population raster | 2.5 arcminutes |
|  | Rec_boating | pleasure craft boating | 1: 40,000 |
|  | Recreational_Crab | recreational crab fishing polygons | 1: 70-120 000 |
|  | RecreationaL_Finfish | recreational finfish polygons | 1: 70-120 000 |
|  | Recreational_Groundfish | recreational groundfish polygons | 1: 70-120 000 |
|  | Recreational_Shrimp | recreational shrimp polygons | 1: 70-120 000 |
|  | SRTM_DEM | SRTM topography data | 7.5 arc second |
| Coastal Protection | coastal_protection | shoreline protected by vegetation |  |
|  | coastal_protection_veg | eelgrass and kelp within 500/1500 m of shore |  |
|  | cs_protect | finished ES data |  |
|  | Kelp_2 | kelp input for coastal protection | 1: 40,000 |
|  | Shorezone | GeoBC shorezone database | 1: 20,000 |
|  | Dunes_3 | used in protection ES model. Limited extent | 1: 20,000 |
|  | Eelgrass_1 | eelgrass input for coastal protection | 1: 40 000 |
| Commercial Demersal Fishing | Roe_fishery | roe fishery | not listed |
|  | crab_fishery | crab fishery | not listed |
|  | geoduck_fishery | geoduck fishery | not listed |
|  | groundfish_fishery | groundfish fishery | not listed |
|  | octopi_fishery | octopus fishery | not listed |
|  | prawn_fishery | prawn fishery | not listed |
|  | scallop_fishery | scallop fishery | not listed |
|  | cucumber_fishery | sea cucumber fishery | not listed |
|  | shrimp_fishery | shrimp fishery | not listed |
|  | squid_fishery | squid fishery | not listed |
|  | urchin_fishery | urchin fishery | not listed |
| Commercial Pelagic Fishery | sablefish_fishery | sablefish fishery | not listed |
|  | anchovy_fishery | anchovy fishery | not listed |
|  | salmon_fishery | salmon fishery | not listed |
|  | herring_fishery | herring fishery | not listed |
| Coastal Recreation | Divesites_BCMCA | dive sites | 1 :40,000 |
|  | Kayak_BCMCA | kayak routes | 1: 40,000 |
|  | Kayak_points | kayak routes in point form |  |
|  | Recreational_fisheries | recreational fisheries | not listed |
|  | Marinas | point file of marinas | 1: 40,000 |
| Energy | Fetch_cmb | produced with InVEST fetch tool |  |
|  | Tidal_energy | areas of interest for future tidal energy dev. | 1: 700-900 000 |
|  | Wave_energy | areas of interest for future wave energy dev. | 1: 700-900 000 |
|  | WaveWatchIII | Wave watch III data | 1 x 1.25 degree |
| Finfish aquaculture | Finfish_aquaculture | finfish aqua. and associated structures | not listed |
| Shellfish aquaculture | Shellfish_aquaculture | shellfish aqua. and associated structures | 1: 40,000 |
| General modeling variables | Can_EEZ | Exclusive economic zone of Canada | 1: 1, 000 000 |
|  | Land_BC | land from global land polygon |  |
|  | Continental_shelf | continental shelf off of BC coast |  |
|  | EEZ_Bathymetry | bathymetry raster. Low res at greater depths | 100 m |
