# Supplementary material for: Mapping cumulative impacts to coastal ecosystem services in British Columbia
Source: PLoS One. 2020 May 4;15(5):e0220092. doi: 10.1371/journal.pone.0220092 (PMC7197858; doi:10.1371/journal.pone.0220092)
Supplement: S3 Table — Note that some category descriptions here describe multiple human activity and stressor data layers (from S2 Table). They are grouped to avoid repetition. (DOCX) [file pone.0220092.s003.docx]

S3 Table . Descriptions of drivers provided to experts to assess risk. Note that some category descriptions here describe multiple driver data layers (from Table S2). They are grouped to avoid repetition

| **Driver Type** | **Description** |
| --- | --- |
| **Fishing (used for all four fisheries)** | One fishing vessel of the specific fishing type indicated and its associated risks such as catch, bycatch and lost fishing gear |
| **Finfish Aquaculture** | One aquaculture facility and its associated effects such as disease and nutrient transmission |
| **Shellfish Aquaculture** | One aquaculture facility including its risks to intertidal habitats and risks of invasive species release |
| **Large Boat Traffic** | One large boat or ship and associated risks such as strikes/collisions, acoustic impacts, and illegal dumping of oil wastes and greywater |
| **Ports, Marinas and Harbours** | One marina/port/harbour, including the dredging required to create it and associated risks of contaminants and breakwaters |
| **Small Docks, Ramps, and Wharves** | One dock/ramp/wharf, and associated risks such as shading and contaminants |
| **Log Dumping, Handling and Storage** | One log-dumping site and associated risks such as scouring and leachates |
| **Ocean Dumping** | One dump event in a site designated for ocean dumping of nontoxic materials |
| **Industry** | One industrial building (e.g. factory) including associated risks such as pollutants |
| **Pulp and Paper** | One pulp mill and associated risks including toxic effluent and leaks |
| **Onshore Mining** | One mining pit and associated risks from discharge and drainage |
| **Human Settlements (including towns and lodges)** | One human dwelling and its associated risks such as pollutants |
| **Agriculture and Forestry** | One farm and its associated risks including silt and pesticide runoff, or one forest cutblock and associated risks including sediment runoff |
| **Climate Change stressors (includes SST stress, UV, and Ocean Acidification)** | For each specific stressor, consider an event to cover the entire phenomenon of climate change and only the current resulting effects |
| **Potential Risk: Future Climate Change** | For each specific stressor, consider an event to cover the entire phenomenon of climate change with a 3 °C  in temperature and a decrease of 0.3 in ocean pH (corresponding to projections for 2100) |
| **Potential Risk: Oil Spill** | One large oil spill (>40 000 m^3^ of oil spilled, approximately the size of the Exxon Valdez oil spill in 1989) |
