# Supplementary material for: Mapping cumulative impacts to coastal ecosystem services in British Columbia
Source: PLoS One. 2020 May 4;15(5):e0220092. doi: 10.1371/journal.pone.0220092 (PMC7197858; doi:10.1371/journal.pone.0220092)
Supplement: S4 Table — (DOCX) [file pone.0220092.s004.docx]

S4 Table . Descriptions of exposure criteria given to experts to assess risk

| **Dimension** | **Description** |
| --- | --- |
| **Area of Influence** | *The spatial influence of a single event of an activity on the area where a service is provided, where a risk may be direct or indirect, measured in kilometers squared (km^2^)*  This represents the impact of a single event of an activity, not the aggregate or cumulative presence of the activity across the seascape. For example, trawling, in total, may impact thousands of square kilometres, but a single trawling event may cover less than 1 to 10 km^2^. It is the latter in which we are interested. If onshore mining negatively impacts the view of an entire bay, then the spatial influence of onshore mining to aesthethic quality is the area of the entire bay.   **Unit of Measurement:**Kilometres squared (km^2^) |
| **Frequency** | *The average annual frequency of individual events of an activity at a particular location, measured in days per year.*  Frequency is not a measure of duration, but rather, how many times an activity occurs in an area in a given year. For example, if fishing groundfish occurs everywhere in a region, but on average only occurs at a given location three times a year, the frequency would be 3 days/yr. Duration will be captured by "Recovery Time". In cases were an activity is ongoing (i.e. there is no pinpoint "event") **the frequency of that activity should be counted as every day** - **365 days/yr**. Fractions represent frequencies less than once per year (e.g. 0.1 days/yr represents once per decade).   **Unit of Measurement:** Days per year (days/yr) |
| **Recovery Time** | *The average time required for the affected ES to return to its former level of provision, following disturbance by a given activity, measured in years.*  Fractions represent times shorter than a single year. Recovery of the ES is related to the resilience of the system to a type of risk. It relates to recovery of a system within a site as well as the necessary species or environmental components that make up the biophysical producers/production of an ES from surrounding areas to recolonize a site. "Biophysical producers" indicates the specific species responsible for producing the ES under consideration.    **Unit of Measurement:** Years (yrs) |
