# Supplementary material for: Mapping cumulative impacts to coastal ecosystem services in British Columbia
Source: PLoS One. 2020 May 4;15(5):e0220092. doi: 10.1371/journal.pone.0220092 (PMC7197858; doi:10.1371/journal.pone.0220092)
Supplement: S5 Table — (DOCX) [file pone.0220092.s005.docx]

S5 Table. Descriptions of consequence criteria given to experts to assess risk

| **Dimension** | **Description** |
| --- | --- |
| **Magnitude of Risk** | *The degree to which an ES is at risk due to potential negative impacts of an activity on the biophysical producers/production of that ES, measured in percent (%).*  For renewable energy, consider the risk to potential renewable energy generated by wind or waves; for fishery-related ES, consider the risk to fishery yield; for marine carbon sequestration, consider the change in the amount of carbon sequestered; for coastal protection, consider the change in erosion risk. Magnitude of risk to ES affected by an activity also addresses the diversity of producers responsible for the ES. If a risk affects a high proportion of ES producers, that ES is likely more vulnerable and less resistant to the risk.   **Unit of Measurement:** Percent (%)  **Guidelines:** 0% = No risk to ES producers or production 100% = Complete loss of ES producers or production |
| **Community Extent** | *The extent of risk on the underlying ecological community responsible for producing an ES, measured in a 0-3 score.*  Human activities can affect the primary biophysical producers of ES directly or indirectly through associated species and habitats. More extensive risks may also affect primary biophysical producers of ES and their associated species and habitats.  For example, shipping would score a 1 for marine recreation if considering that ship-strikes impact sought-after marine mammals. Fishing however, would score a 2 if marine mammal abundance and distribution are at risk indirectly through a decreased prey base. Larger phenomena such as climate change could rank 3 for altering coastal habitat and thereby affecting the abundance of prey species.   **Unit of Measurement:** Integer from 0 to 3  0 = An activity might occur on some part of the region, but does not affect the biophysical producers of the ES  1 = The activity affects the biophysical producers of the ES (directly or indirectly)  2 = The activity affects the biophysical producers and supporting species  3 = The activity affects the biophysical producers, supporting species and surrounding habitat structure |
| **Access to Service** | *The change in the ability and rights of people to access an area so that they may benefit from the ES in question, measured as percent (%)*  For example, if log dumping and handling restricts access to subsistence fishing in an area by 50%, than this would be scored as 50. If it completely closes an area that people use to fish, than this would be scored as 100.   **Unit of Measurement:** Percent (%)  **Guidelines:** 0% = Positive or no effect on the accessibility of the ES 100% = Complete loss of access to the ES |
| **Quality of Service** | *The change in the enjoyment or benefit that people derive from an ES given the same quantity of good or experience, measured as percent (%)*  Assuming that there are no limitations in accessibility, evaluate how an activity would affect the enjoyment of that service. This is intended to capture the risk of human activities to the **intangible benefits** that people derive from ES. For landscape aesthetics and recreation, consider the change in the quality of the scenery or enjoyment. For fisheries and aquaculture, if agricultural runoff reduces the water quality of an area such that shellfish harvest poses a major health hazard, this would be scored as a 100.   **Unit of Measurement:** Percent (%)  **Guidelines:** 0% = Positive or no effect on the quality of service 100% = Complete loss of enjoyment or benefit provided by the ES |
