# Supplementary material for: Mapping cumulative impacts to coastal ecosystem services in British Columbia
Source: PLoS One. 2020 May 4;15(5):e0220092. doi: 10.1371/journal.pone.0220092 (PMC7197858; doi:10.1371/journal.pone.0220092)
Supplement: S6 Table — Values represent means and standard errors in brackets. (DOCX) [file pone.0220092.s006.docx]

S6 Table . Normalized expert scores for the seven risk criteria of different drivers on the ecosystem services. Values represent means and standard errors in brackets.

| **Aesthetics (n = 4)** | Area of Influence | Frequency | Recovery Time | Magnitude | Community | Access | Quality |
| --- | --- | --- | --- | --- | --- | --- | --- |
| Demersal Destructive | 0.00383 (0.00308) | 0.0128 (0.00456) | 0.04 (0.02) | 0.03 (0.01) | 0 (0) | 0 (0) | 0.1 (0.02) |
| Demersal non-destructive low bycatch | 0.00383 (0.003078) | 0.0128 (0.00456) | 0.015 (0.005) | 0.03 (0.01) | 0 (0) | 0 (0) | 0.1 (0.02) |
| Demersal non-destructive high bycatch | 0.00383 (0.003078) | 0.0128 (0.00456) | 0.03 (0.01) | 0.03 (0.01) | 0 (0) | 0 (0) | 0.1 (0.02) |
| Pelagic Low Bycatch | 0.00217 (0.00142) | 0.0128 (0.00456) | 0.0101 (0.0099) | 0.03 (0.01) | 0 (0) | 0 (0) | 0.1 (0.02) |
| Pelagic high bycatch | 0.00217 (0.00142) | 0.0128 (0.00456) | 0.0501 (0.0499) | 0.03 (0.01) | 0 (0) | 0 (0) | 0.1 (0.02) |
| Recreational Fishing | 0.000667 (0.000333) | 0.0785 (0.0432) | 0.0051 (0.0049) | 0.03 (0.01) | 0 (0) | 0 (0) | 0.03 (0.02) |
| Finfish Aquaculture | 0.00145 (0.00119) | 0.501 (0.288) | 0.0466 (0.0291) | 0.015 (0.0075) | 0 (0) | 0 (0) | 0.03 (0.02) |
| Shellfish Aquaculture | 0.0015 (0.00117) | 0.501 (0.288) | 0.00733 (0.00636) | 0.015 (0.0075) | 0 | 0 (0) | 0.03 (0.02) |
| Large Boat Traffic | 0.0075 (0.0025) | 0.501 (0.498) | 0.06 (0.06) | 0.15 (0.0075) | 0 (0) | 0 (0) | 0.2 (0.02) |
| Ports, Marinas, and harbours | 0.00175 (0.000854) | 0.501 (0.288) | 0.0383 (0.0311) | 0.1 (0.05) | 1 (0) | 0 (0) | 0.2 (0.02) |
| Small docks, ramps, wharfs | 0.000675 (0.00035) | 0.503 (0.286) | 0.005 (0.00288) | 0.015 (0.0075) | 0.333 (0) | 0 (0) | 0.05 (0.02) |
| Log dumping, handling, storage | 0.00125 (0.000479) | 0.319 (0.2366) | 0.353 (0.323) | 0.375 (0.125) | 0.666 (0) | 0 (0) | 0.5 (0.02) |
| Ocean dumping | 0.0028 (0.00241) | 0.257 (0.247) | 0.336 (0.331) | 0.075 (0.036) | 0 (0) | 0 (0) | 0.5 (0.02) |
| Industry | 0.01 (0.00578) | 0.334 (0.302) | 0.05 (0.002) | 0.375 (0.122) | 1 (0) | 0 (0) | 0.5 (0.02) |
| Pulp and Paper | 0.01 (0.00577) | 0.334 (0.302) | 0.05 (0.002) | 0.375 (0.122) | 1 (0) | 0 (0) | 0.7 (0.02) |
| Onshore Mining | 0.01 (0.00577) | 0.334247 (0.302) | 0.05 (0.002) | 0.375 (0.122) | 0.666 (0) | 0 (0) | 0.7 (0.02) |
| Human settlements | 0.0183 (0.0159) | 0.342 (0.302) | 0.2 (0.1) | 0.375 (0.122) | 0.666 (0) | 0 (0) | 0.3 (0.02) |
| Agriculture | 0.01 (0.00577) | 0.342 (0.302) | 0.1 (0.05) | 0.375 (0.122) | 1 (0) | 0 (0) | 0.2 (0.02) |
| Ocean acidification | 0.0025 (0.00122) | 1 (0) | 0.2 (0) | 0 (0) | 0 (0) | 0 (0) | 0 (0) |
| Sea Level Rise | 0.0025 (0.00122) | 1 (0) | 0.2 (0) | 0 (0) | 0 (0) | 0 (0) | 0 (0) |
| Sea temp change | 0.0025 (0.00122) | 1 (0) | 0.2 (0) | 0 (0) | 0 (0) | 0 (0) | 0 (0) |
| UV change | 0.0025 (0.00122) | 1 (0) | 0.2 (0) | 0 (0) | 0 (0) | 0 (0) | 0 (0) |
| Future Sea temp change | 0.005 (0.0025) | 1 (0) | 1 (0) | 0 (0) | 0 (0) | 0 (0) | 0 (0) |
| Future ocean acidification | 0.005 (0.0025) | 1 (0) | 1 (0) | 0 (0) | 0 (0) | 0 (0) | 0 (0) |
| Future oil spill | 0.51 (0.4) | 0.50137 (0.45) | 0.16 (0.14) | 1 (0) | 0.5 (0.167) | 0 (0) | 0.95 (0) |
| **Coastal Protection (n = 3)** | Area of Influence | Frequency | Recovery Time | Magnitude | Community | Access | Quality |
| Demersal Destructive | 0 (0) | 0 (0) | 0 (0) | 0 (0) | 0 (0) | 0 (0) | 0 (0) |
| Demersal non-destructive low bycatch | 0 (0) | 0 (0) | 0 (0) | 0 (0) | 0 (0) | 0 (0) | 0 (0) |
| Demersal non-destructive high bycatch | 0 (0) | 0 (0) | 0 (0) | 0 (0) | 0 (0) | 0 (0) | 0 (0) |
| Pelagic Low Bycatch | 0 (0) | 0 (0) | 0 (0) | 0 (0) | 0 (0) | 0 (0) | 0 (0) |
| Pelagic high bycatch | 0 (0) | 0 (0) | 0 (0) | 0 (0) | 0 (0) | 0 (0) | 0 (0) |
| Recreational Fishing | 0.207 (0.115) | 0.0411 (0.012) | 0.215 (0.0623) | 0.1 (0.0763) | 0.333(0) | 0.1 (0.0765) | 0.1 (0.0763) |
| Finfish Aquaculture | 0 (0) | 0 (0) | 0 (0) | 0 (0) | 0 (0) | 0 (0) | 0 (0) |
| Shellfish Aquaculture | 0 (0) | 0 (0) | 0 (0) | 0 (0) | 0 (0) | 0 (0) | 0 (0) |
| Large Boat Traffic | 0.075 (0.025) | 0.178 (0.0959) | 0.0755 (0.0745) | 0.5 (0) | 0.666 (0) | 0.1 (0.0765) | 0.25 (0.0577) |
| Ports, Marinas, and harbours | 0.01 (0) | 0.137 (0.075) | 0.3 (0.12) | 0.2 (0.06) | 0.333 (0) | 0.1 (0) | 0.25 (0.1) |
| Small docks, ramps, wharfs | 0.001 (0.00053) | 0.0274 (0.0137) | 0.15 (0.05) | 0.1 (0.05) | 0.333 (0) | 0.1 (0.05) | 0.25 (0.05) |
| Log dumping, handling, storage | 0.05 (0) | 0.0274 (0.0137) | 0.3 (0.1) | 0.5 (0.25) | 0.666 (0.192) | 0.25 (0.05) | 0.5 (0.25) |
| Ocean dumping | 0.05 (0.045) | 0.00274 (0.0137) | 0.025 (0.0125) | 0 (0) | 0 (0) | 0 (0) | 0 (0) |
| Industry | 0.01 (0.005) | 0.0137 (0.00791) | 0.15 (0.05) | 0.5 (0.25) | 0.666 (0.192) | 0.25 (0.05) | 1 (0) |
| Pulp and Paper | 0.01 (0.005) | 0.0274 (0.0137) | 0.3 (0.1) | 0.5 (0.25) | 0.666 (0.192) | 0.5 (0.15) | 1 (0) |
| Onshore Mining | 0.01 (0.005) | 0.00548 (0.000913) | 0.1 (0.05) | 0.25 (0.05) | 0.666 (0.192) | 0.25 (0.05) | 1 (0) |
| Human settlements | 0.1 (0.005) | 0.0685 (0.0158) | 0.5 (0.25) | 0.5 (0.25) | 0.666 (0.192) | 0.5 (0.25) | 1 (0) |
| Agriculture | 0.05 (0.045) | 0.0137 (0.00791) | 0.25 (0.1) | 0.5 (0.15) | 0.666 (0.192) | 0.5 (0.2) | 1 (0) |
| Ocean acidification | 0 (0) | 0 (0) | 0 (0) | 0 (0) | 0 (0) | 0 (0) | 0 (0) |
| Sea Level Rise | 1 (0) | 0.501 (0.144) | 0.75 (0.25) | 0.75 (0.25) | 1 (0) | 1 (0) | 1 (0) |
| Sea temp change | 0 (0) | 0 (0) | 0 (0) | 0 (0) | 0 (0) | 0 (0) | 0 (0) |
| UV change | 0 (0) | 0 (0) | 0 (0) | 0 (0) | 0 (0) | 0 (0) | 0 (0) |
| Future Sea temp change | 0 (0) | 0 (0) | 0 (0) | 0 (0) | 0 (0) | 0 (0) | 0 (0) |
| Future ocean acidification | 0 (0) | 0 (0) | 0 (0) | 0 (0) | 0 (0) | 0 (0) | 0 (0) |
| Future oil spill | 0.1 (0.05) | 0.000822 (0.00041) | 0.25 (0.1) | 0.1 (0.05) | 0.666 (0.192) | 0.5 (0.2) | 1 (0) |
| **Energy Potential (n = 1)** | Area of Influence | Frequency | Recovery Time | Magnitude | Community | Access | Quality |
| Demersal Destructive | 0.2 | 0.002739726 | 1 | 0.2 | 0.666666667 | 0.5 | 0.5 |
| Demersal non-destructive low bycatch | 0.2 | 0.002739726 | 1 | 0.2 | 0.666666667 | 0.5 | 0.5 |
| Demersal non-destructive high bycatch | 0.2 | 0.002739726 | 1 | 0.2 | 0.666666667 | 0.5 | 0.5 |
| Pelagic Low Bycatch | 0.2 | 0.002739726 | 1 | 0.2 | 0.666666667 | 0.5 | 0.5 |
| Pelagic high bycatch | 0.2 | 0.002739726 | 1 | 0.2 | 0.666666667 | 0.5 | 0.5 |
| Recreational Fishing | 0.2 | 0.002739726 | 1 | 0.1 | 0.666666667 | 0 | 0 |
| Finfish Aquaculture | 0.2 | 0.002739726 | 1 | 0 | 0 | 0 | 0 |
| Shellfish Aquaculture | 0 | 0 | 0 | 0 | 0 | 0 | 0 |
| Large Boat Traffic | 0.2 | 0 | 0 | 0.5 | 0.333333333 | 0.75 | 0.75 |
| Ports, Marinas, and harbours | 1 | 0 | 0 | 0 | 0 | 0.5 | 0.25 |
| Small docks, ramps, wharfs | 1 | 0 | 0 | 0 | 0 | 0 | 0 |
| Log dumping, handling, storage | 0 | 0 | 0 | 0 | 0 | 0 | 0 |
| Ocean dumping | 0.2 | 0 | 0 | 0.25 | 0.333333333 | 1 | 0.5 |
| Industry | 1 | 0.002739726 | 1 | 0.25 | 0.333333333 | 0 | 0 |
| Pulp and Paper | 0 | 0 | 0 | 0 | 0 | 0 | 0 |
| Onshore Mining | 0 | 0 | 0 | 0 | 0 | 0 | 0 |
| Human settlements | 1 | 0.002739726 | 1 | 0 | 0 | 0 | 0 |
| Agriculture | 0 | 0 | 0 | 0 | 0 | 0 | 0 |
| Ocean acidification | 0.04 | 0.002739726 | 1 | 0 | 0 | 0 | 0 |
| Sea Level Rise | 0.04 | 0.002739726 | 1 | 0 | 0 | 0 | 0 |
| Sea temp change | 0.04 | 0.002739726 | 1 | 0 | 0 | 0 | 0 |
| UV change | 0.04 | 0.002739726 | 1 | 0 | 0 | 0 | 0 |
| Future Sea temp change | 0.04 | 0.002739726 | 1 | 0 | 0 | 0 | 0 |
| Future ocean acidification | 0.04 | 0.002739726 | 1 | 0 | 0 | 0 | 0 |
| Future oil spill | 0.04 | 0.002739726 | 1 | 0.5 | 0.333333333 | 0.5 | 0.5 |
| **Benefits from Aquaculture (n = 7)** | Area of Influence | Frequency | Recovery Time | Magnitude | Community | Access | Quality |
| Demersal Destructive | 0.00005 (0) | 0.00137 (0.000685) | 0.0001 (0.00005) | 0.233 (0.145) | 0.555 (0.294) | 0.05 (0.02) | 0.05 (0.02) |
| Demersal non-destructive low bycatch | 0.00005 (0) | 0.00137 (0.000685) | 0.0001 (0.00005) | 0.266 (0.145) | 0.555 (0.294) | 0 (0) | 0 (0) |
| Demersal non-destructive high bycatch | 0.00005 (0) | 0.00137 (0.000685) | 0.0001 (0.00005) | 0.233 (0.145) | 0.555 (0.294) | 0 (0) | 0 (0) |
| Pelagic Low Bycatch | 0.00337 (0.00168) | 0.220 (0.118) | 0.00673 (0.00331) | 0.275 (0.111) | 0.5 (0.215) | 0 (0) | 0.05 (0.02) |
| Pelagic high bycatch | 0.00005 (0) | 0 (0) | 0 (0) | 0.266 (0.145) | 0.555 (0.294) | 0 (0) | 0 (0) |
| Recreational Fishing | 0.204 (0.118) | 0 (0) | 0.202 (0.0735) | 0.2 (0.075) | 0.444 (0.293) | 0 (0) | 0 (0) |
| Finfish Aquaculture | 0.00173 (0.000815) | 0.666 (0.333) | 0.246 (0.103) | 0.49 (0.181) | 0.666 (0.211) | 0.166 (0.0752) | 0.416 (0.220) |
| Shellfish Aquaculture | 0.00055 (0.00022) | 1 (0) | 0.003 (0.001) | 0.283 (0.211) | 0.444 (0.294) | 0 (0) | 0 (0) |
| Large Boat Traffic | 0.1 (0.02) | 1 (0) | 0.0002 (0.0001) | 0.2 (0.0577) | 0.555 (0.222) | 0 (0) | 0.1 (0) |
| Ports, Marinas, and harbours | 0.1 (0.02) | 1 (0) | 0.0002 (0.0001) | 0.166 (0.0333) | 0.555 (0.111) | 0.01 (0) | 0.1 (0) |
| Small docks, ramps, wharfs | 0.0001 (0.00022) | 1 (0) | 0.006 (0.00310 | 0.133 (0.0333) | 0.444 (0.111) | 0.001 (0) | 0.1 (0) |
| Log dumping, handling, storage | 0 (0) | 0 (0) | 0 (0) | 0.05 (0.0288) | 0.333 (0.192) | 0 (0) | 0 (0) |
| Ocean dumping | 0 (0) | 0 (0) | 0 (0) | 0.075 (0.0478) | 0.416 (0.25) | 0 (0) | 0 (0) |
| Industry | 0.1 (0.02) | 1 (0) | 0.006 (0.0034) | 0.183 (0.0726) | 0.555 (0.111) | 0.001 (0) | 0.1 (0) |
| Pulp and Paper | 0.1 (0.02) | 1 (0) | 0.006 (0.0034) | 0.316 (0.136) | 0.666 (0.192) | 0.01 (0) | 0 (0) |
| Onshore Mining | 0.1 (0.02) | 1 (0) | 0.006 (0.0034) | 0.283 (0.130) | 0.777 (0.111) | 0.005 (0.0021) | 0.1 (0.045) |
| Human settlements | 0.1 (0.02) | 1 (0) | 0.006 (0.0034) | 0.383 (0.262) | 0.777 (0.111) | 0.355 (0.145) | 0.35 (0.156) |
| Agriculture | 0.1 (0.02) | 1 (0) | 0.006 (0.0034) | 0.35 (0.175) | 0.666 (0.192) | 0.15 (0.092) | 0.35 (0.156) |
| Ocean acidification | 0.55 (0.245) | 0.534 (0.265) | 0.501 (0.199) | 0.725 (0.131) | 1 (0) | 0 (0) | 0.475 (0.325) |
| Sea Level Rise | 0.055 (0.245) | 0.503 (0.297) | 0.2 (0.099) | 0.227 (0.109) | 0.75 (0.159) | 0.125 (0.075) | 0.1 (0) |
| Sea temp change | 0.055 (0.245) | 0.55 (0.245) | 0.202 (0.098) | 0.6 (0.204) | 0.916 (0.0833) | 0.1 (0.033) | 0.125 (0.075) |
| UV change | 0.055 (0.245) | 0.55 (0.245) | 0.202 (0.098) | 0.1 (0.0664) | 0.222 (0.111) | 0 (0) | 0 (0) |
| Future Sea temp change | 0.55 (0.45) | 0.705 (0.294) | 0.205 (0.195) | 0.85 (0.195) | 1 (0) | 0.4 (0.4) | 0.45 (0.35) |
| Future ocean acidification | 0.55 (0.45) | 0.842 (0.157) | 0.503 (0.407) | 0.975 (0.025) | 1 (0) | 0.45 (0.35) | 0.5 (0.4) |
| Future oil spill | 0.055 (0.45) | 0.0000151 (0.0000123) | 0.035 (0.005) | 0.561 (0.168) | 0.933 (0.0666) | 0.666 (0.333) | 0.9 (0.0577) |
| **Benefits from Fisheries (n = 17)** | Area of Influence | Frequency | Recovery Time | Magnitude | Community | Access | Quality |
| Demersal Destructive | 0.000808 (0.000403) | 0.348 (0.114) | 0.000804 (0.000457) | 0.695 (0.111) | 0.962 (0.0371)) | 0.45 (0.139) | 0.411 (0.149) |
| Demersal non-destructive low bycatch | 0.000692 (0.000269) | 0.362 (0.123) | 0.0000421 (0.0000164) | 0.251 (0.0759) | 0.666 (0.0962) | 0.206 (0.0771) | 0.195 (0.0742) |
| Demersal non-destructive high bycatch | 0.00123 (0.000886) | 0.363 (0.123) | 0.0007102 (0.000486) | 0.601 (0.123) | 0.888 (0.0555) | 0.393 (0.126) | 0.525 (0.112) |
| Pelagic Low Bycatch | 0.00264 (0.00118) | 0.283 (0.102) | 0.000063 (0.0000185) | 0.228 (0.0741) | 0.518 (0.0979) | 0.137 (0.0617) | 0.163 (0.0613) |
| Pelagic high bycatch | 0.00264 (0.00118) | 0.308 (0.109) | 0.000235 (0.0000963) | 0.506 (0.0962) | 0.814 (0.0585) | 0.406 (0.142) | 0.425 (0.106) |
| Recreational Fishing | 0.000363 (0.000183) | 0.248 (0.0925) | 0.0000591 (0.0000205) | 0.306 (0.106) | 0.592 (0.0925) | 0.106 (0.0291) | 0.17 (0.0614) |
| Finfish Aquaculture | 0.00861 (0.00831) | 0.916 (0.0833) | 0.00032 (0.000108) | 0.495 (0.107) | 0.703 (0.129) | 0.622 (0.125) | 0.511 (0.145) |
| Shellfish Aquaculture | 0.000176 (0.0000937) | 0.909 (0.0909) | 0.0000975 (0.0000253) | 0.361 (0.119) | 0.625 (0.146) | 0.45 (0.132) | 0.307 (0.121) |
| Large Boat Traffic | 0.00281 (0.00126) | 0.629 (0.120) | 0.000483 (0.000451) | 0.158 (0.0598) | 0.566 (0.0867) | 0.176 (0.0956) | 0.245 (0.0981) |
| Ports, Marinas, and harbours | 0.000140 (0.0000805) | 0.865 (0.0923) | 0.000687 (0.000401) | 0.247 (0.0911) | 0.833 (0.0745) | 0.267 (0.125) | 0.277 (0.138) |
| Small docks, ramps, wharfs | 0.0000391 (0.0000179) | 0.826 (0.0957) | 0.000095 (0.0000386) | 0.181 (0.0872) | 0.8 (0.0888) | 0.184 (0.106) | 0.187 (0.119) |
| Log dumping, handling, storage | 0.000135 (0.0000832) | 0.863 (0.0977) | 0.000316 (0.0000988) | 0.3244 (0.121) | 0.741 (0.107) | 0.418 (0.135) | 0.555 (0.147) |
| Ocean dumping | 0.00169 (0.00111) | 0.406 (0.135) | 0.000426 (0.000113) | 0.527 (0.122) | 0.833 (0.0895) | 0.287 (0.128) | 0.541 (0.134) |
| Industry | 0.000807 (0.000403) | 0.348 (0.114) | 0.00106 (0.000604) | 0.623 (0.121) | 0.925 (0.0489) | 0.572 (0.155) | 0.65 (0.137) |
| Pulp and Paper | 0.00302 (0.00117) | 0.909 (0.0909) | 0.000954 (0.000433) | 0.473 (0.127) | 0.933 (0.0444) | 0.542 (0.142) | 0.725 (0.111) |
| Onshore Mining | 0.00225 (0.00129) | 0.9 (0.1) | 0.00211 (0.00111) | 0.36 (0.117) | 0.851 (0.0585) | 0.428 (0.151) | 0.451 (0.156) |
| Human settlements | 0.0128 (0.0088) | 0.916 (0.0833) | 0.00192 (0.000913) | 0.49 (0.106) | 0.925 (0.0489) | 0.26 (0.0682) | 0.35 (0.0916) |
| Agriculture | 0.00135 (0.000875) | 0.701 (0.0987) | 0.000681 (0.000440) | 0.252 (0.0937) | 0.866 (0.0737) | 0.316 (0.107) | 0.386 (0.135) |
| Ocean acidification | 0.295 (0.121) | 0.749 (0.131) | 0.103 (0.0996) | 0.646 (0.134) | 0.833 (0.113) | 0.338 (0.122) | 0.384 (0.132) |
| Sea Level Rise | 0.305 (0.118) | 0.818 (0.121) | 0.124 (0.109) | 0.484 (0.165) | 0.777 (0.124) | 0.145 (0.0921) | 0.207 (0.119) |
| Sea temp change | 0.297 (0.121) | 0.732 (0.138) | 0.113 (0.111) | 0.644 (0.142) | 0.851 (0.112) | 0.306 (0.0908) | 0.351 (0.121) |
| UV change | 0.4 (0.151) | 0.709 (0.146) | 0.113 (0.111) | 0.54 (0.153) | 0.888 (0.111) | 0.0825 (0.0348) | 0.193 (0.0746) |
| Future Sea temp change | 0.427 (0.146) | 0.713 (0.146) | 0.147 (0.122) | 0.861 (0.122) | 0.962 (0.0371) | 0.618 (0.0981) | 0.475 (0.133) |
| Future ocean acidification | 0.427 (0.146) | 0.801 (0.133) | 0.147 (0.122) | 0.78 (0.107) | 1 (0) | 0.688 (0.103) | 0.577 (0.119) |
| Future oil spill | 0.0152 (0.00965) | 0.305 (0.136) | 0.000391 (0.0000999) | 0.576 (0.143) | 0.766 (0.1) | 0.641 (0.138) | 0.81 (0.102) |
| **Coastal Recreation (n = 12)** | Area of Influence | Frequency | Recovery Time | Magnitude | Community | Access | Quality |
| Demersal Destructive | 0.00101 (0.000998) | 0.208 (0.0863) | 0.00046 (0.000207) | 0.542 (0.120) | 1 (0) | 0.417 (0.295) | 0.6 (0.264) |
| Demersal non-destructive low bycatch | 0.00100 (0.000998) | 0.209 (0.0853) | 0.000245 (0.000157) | 0.326 (0.149) | 0.4 (0.163) | 0.0933 (0.0783) | 0.283 (0.130) |
| Demersal non-destructive high bycatch | 0.0100 (0.00999) | 0.209 (0.116) | 0.000413 (0.000189) | 0.428 (0.154) | 0.733 (0.124) | 0.183 (0.158) | 0.533 (0.260) |
| Pelagic Low Bycatch | 0.00501 (0.00500) | 0.211 (0.115) | 0.000094 (0.0000541) | 0.172 (0.0934) | 0.2 (0.0816) | 0.09 (0.08) | 0.27 (0.142) |
| Pelagic high bycatch | 0.00501 (0.00499) | 0.211 (0.115) | 0.00036 (0.000456) | 0.288 (0.119) | 0.466 (0.133) | 0.19 (0.155) | 0.506 (0.283) |
| Recreational Fishing | 0.123 (0.0814) | 0.404 (0.150) | 0.101 (0.0512) | 0.134 (0.0956) | 0.4 (0.194) | 0.05 (0.05) | 0.75 (0.25) |
| Finfish Aquaculture | 0.00110 (0.000970) | 0.887 (0.113) | 0.000393 (0.000137) | 0.4 (0.151) | 0.733 (0.194) | 0.606 (0.298) | 0.55 (0.256) |
| Shellfish Aquaculture | 0.000809 (0.000797) | 0.878 (0.121) | 0.000335 (0.000151) | 0.275 (0.118) | 0.666 (0.172) | 0.502 (0.236) | 0.305 (0.201) |
| Large Boat Traffic | 0.00814 (0.00796) | 0.738 (0.169) | 0.000102 (0.0000416) | 0.186 (0.0635) | 0.611 (0.159) | 0.17 (0.0851) | 0.406 (0.259) |
| Ports, Marinas, and harbours | 0.000808 (0.000798) | 1 (0) | 0.000955 (0.000441) | 0.42 (0.146) | 0.888 (0.111) | 0.425 (0.375) | 0.5 (0.4) |
| Small docks, ramps, wharfs | 0.000103 (0.0000991) | 0.909 (0.0904) | 0.000425 (0.000226) | 0.286 (0.131) | 0.722 (0.181) | 0.41 (0.39) | 0.46 (0.44) |
| Log dumping, handling, storage | 0.000134 (0.000133) | 0.887 (0.113) | 0.000925 (0.000394) | 0.26 (0.141) | 0.888 (0.111) | 0.666 (0.241) | 0.4 (0.252) |
| Ocean dumping | 0.000126 (0.0000942) | 0.365 (0.317) | 0.000912 (0.000572) | 0.261 (0.131) | 0.555 (0.164) | 0.266 (0.242) | 0.45 (0.202) |
| Industry | 0.00455 (0.00261) | 0.751 (0.249) | 0.00088 (0.000656) | 0.34 (0.119) | 0.866 (0.0816) | 0.583 (0.281) | 0.6 (0.264) |
| Pulp and Paper | 0.0296 (0.0276) | 0.751 (0.249) | 0.00074 (0.000587) | 0.336 (0.142) | 0.866 (0.0816) | 0.266 (0.167) | 0.55 (0.15) |
| Onshore Mining | 0.000201 (0.000115) | 0.750 (0.249) | 0.00098 (0.000841) | 0.252 (0.171) | 0.833 (0.0962) | 0.273 (0.263) | 0.5 (0.152) |
| Human settlements | 0.00604 (0.00398) | 1 (0) | 0.00224 (0.00194) | 0.458 (0.149) | 0.944 (0.0555) | 0.266 (0.218) | 0.416 (0.136) |
| Agriculture | 0.250 (0.249) | 0.955 (0.0445) | 0.00035 (0.000173) | 0.406 (0.144) | 0.722 (0.102) | 0.173 (0.163) | 0.433 (0.176) |
| Ocean acidification | 0.232 (0.192) | 1 (0) | 0.00206 (0.000802) | 0.155 (0.0931) | 0.733 (0.0666) | 0.14 (0.13) | 0.336 (0.200) |
| Sea Level Rise | 0.0322 (0.0148) | 1 (0) | 0.00176 (0.000917) | 0.258 (0.159) | 0.466 (0.2) | 0.136 (0.131) | 0.24 (0.2) |
| Sea temp change | 0.304 (0.187) | 1 (0) | 0.002 (0.000774) | 0.328 (0.152) | 0.777 (0.0703) | 0.14 (0.13) | 0.306 (0.203) |
| UV change | 0.373 (0.313) | 1 (0) | 0.00203 (0.00151) | 0.118 (0.0776) | 0.555 (0.141) | 0.2 (0.1) | 0.375 (0.325) |
| Future Sea temp change | 0.304 (0.187) | 1 (0) | 0.203 (0.199) | 0.636 (0.199) | 0.944 (0.0555) | 0.4 (0.3) | 0.65 (0.35) |
| Future ocean acidification | 0.304 (0.187) | 1 (0) | 0.0236 (0.0191) | 0.511 (0.157) | 0.888 (0.0702) | 0.566 (0.268) | 0.683 (0.316) |
| Future oil spill | 0.0153 (0.00923) | 0.00199 (0.000768) | 0.000391 (0.000141) | 0.415 (0.174) | 0.888 (0.0702) | 0.673 (0.326) | 0.683 (0.316) |
