# Supplementary material for: Mapping cumulative impacts to coastal ecosystem services in British Columbia
Source: PLoS One. 2020 May 4;15(5):e0220092. doi: 10.1371/journal.pone.0220092 (PMC7197858; doi:10.1371/journal.pone.0220092)
Supplement: S1 Fig — Side by side comparison of impact maps considering all risk criteria, including ecosystem service supply, service, and value (maps on the left) versus only considering biophysical criteria of risk which only assesses impact to ecosystem service supply (maps on the right). Map pairs are for A) aesthetics, B) coastal protection, C) benefits from commercial demersal fisheries, D) benefits from commercial pelagic fisheries, E) coastal recreation, F) potential renewable energy, G) benefits from finfish aquaculture, and H) benefits from shellfish aquaculture. (DOCX) [file pone.0220092.s007.docx]

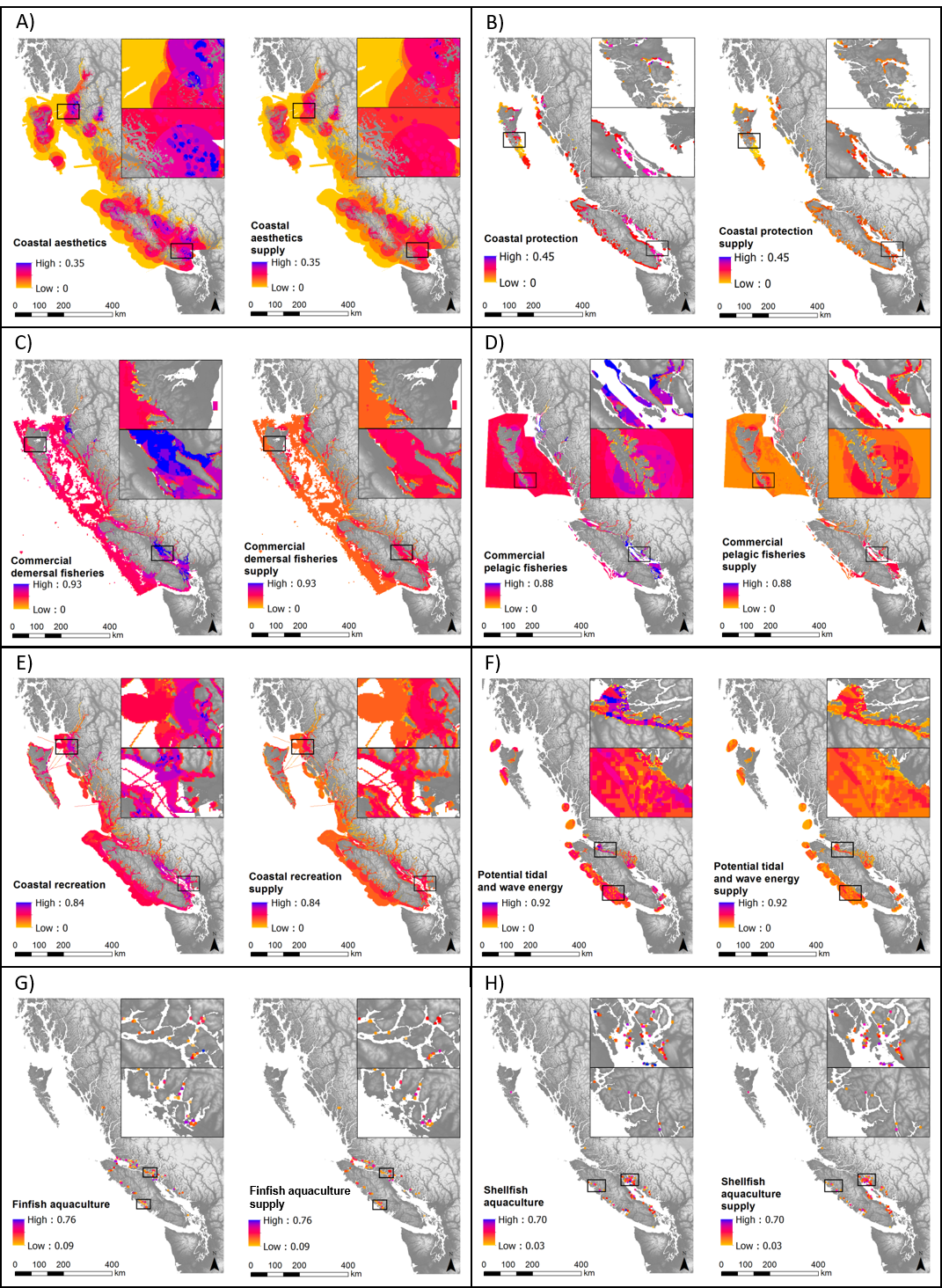


S1 Fig. Side by side comparison of impact maps considering all risk criteria, including ecosystem service supply, service, and value (maps on the left) versus only considering biophysical criteria of risk which only assesses impact to ecosystem service supply (maps on the right). Map pairs are for A) aesthetics, B) coastal protection, C) benefits from commercial demersal fisheries, D) benefits from commercial pelagic fisheries, E) coastal recreation, F) potential renewable energy, G) benefits from finfish aquaculture, and H) benefits from shellfish aquaculture.
