# Supplementary material for: Mapping cumulative impacts to coastal ecosystem services in British Columbia
Source: PLoS One. 2020 May 4;15(5):e0220092. doi: 10.1371/journal.pone.0220092 (PMC7197858; doi:10.1371/journal.pone.0220092)
Supplement: S1 File — (DOCX) [file pone.0220092.s008.docx]

##### Supplementary Methods

Ecosystem Service Models

**Benefits from Aquaculture:**

While the InVEST aquaculture model projects biomass production over time of aquaculture facilities, the data needed to do so were not readily available. Given that only the location of aquaculture facilities was required for this project, the InVEST aquaculture model was unnecessary. Vectors showing the location of shellfish and finfish aquaculture facilities were obtained from GeoBC, and this was sufficient to map the ecosystem service.

**Recreation:**

The InVEST Overlap analysis model was run using the ‘Grid the Seascape’ method, with a 1000 metre grid. The Inputs were kayaking and pleasure craft routes, dive sites, recreational fishing sites, coastal parks, and marinas. The raw data for recreational fishing sites distinguished between different catches, but was combined so as not to bias the output toward recreational fishing. The coastal campsites and marinas points were buffered by 150 and 2000 metres respectively.

**Coastal Protection**:

The Invest Coastal Vulnerability model was run with the following inputs: the shorezone file, described in Table A1 below, with a rank assigned representing erosion risk dependent upon the coastal class of the shorezone data (ranks listed below), natural vegetation layers consisting of dunes, eelgrass, and kelp, a continental shelf layer provided by InVEST, the SRTM DEM from the USGS, and an average depth of 2000 metres. The output produces a raster called “Eros Index” that includes a vulnerability index as well as a ‘protection from vegetation’ score. A coastline segment with no protection from vegetation scores a 5 in this category. Therefore, all cells with scores lower than 5 were extracted, and displayed according to their vulnerability index. Additionally, the natural vegetation scores were determined by the NaturalHabitat.csv table in the vulnerability table. The original values offered by InVEST were used; a protection distance of 1500 m for kelp, 500 m for eelgrass, and 300 metres for dunes. The ‘protection rank’ of dunes, eelgrass, and kelp were 2, 4 and 4, respectively. It should be noted that all three natural vegetation files are incomplete and likely underestimate the extent of the habitats. This is especially true of the dunes layer, which is confined exclusively to the Salish sea.

**Aesthetic Quality:**

The InVEST model functions by determining areas that are visually impacted and delineating the locations from which these areas can be seen. It then grids the area of interest and calculates the number of impacted sites visible for each cell in grid. However, this assumes areas with no impact are visually pleasing, and requires a subjective determination of what constitutes a visual impact. Rather than use this approach, we opted to adapt it by determining the visible extent from areas that are frequented by people, using the recreation inputs from the Overlap model as well as the InVEST provided population data. This assumes that an underlying driver of recreational activities is the view.

The InVEST tool requires point inputs, therefore the kayak, recreational boating, population, and recreational fishing layers had to be converted to points. Recreational fishing, kayak and pleasure craft boating routes were converted using the ‘Create random points’ tool. For the fishing polygons, inputs were 3000 “randomly generated” points, spaced at a minimum of 750 metres apart. The tool works by adding up to the specified amount of points without crossing the minimum distance. However, the model was unable to fit all 3000 points for any of the fishing polygons. This ensures that each point is the minimum 750 m distance from at least one other point, but does not guarantee an equal distance among all points. While imperfect, it does ensure that the viewshed completely includes the fishing polygon, and that the visibility of areas outside of the fishing polygon is determined at approximately 750 metre intervals around the perimeter. The kayak and pleasure craft routes were converted to points with a minimum distance of 1000 metres. The points along a single segment are all ~1000 m apart, and where lines overlap they are close as the minimum distance only applies to points within the same segment. The population data was converted to puts using the ‘Raster to point’ tool. This creates a point for every raster cell. However, only cells that were within a 5 km buffer of the Shorezone coastline were included. In addition, cells with a value of 1 or 0 were excluded (i.e. areas with population densities less than 2 people/25 ha). Camping and dive sites were also included.

The other inputs included the SRTM 7.5 arc-second DEM and a special AOI that was created specifically for this model. The AOI simply followed the contours of the viewshed of an earlier run (with a coarser DEM) to shorten the computing time. The output is a raster that models whether each 500 x 500 m cell visible from one of the input points.

**Potential Renewable Energy**

The Wave Energy model was not completed with InVEST. The British Columbia Marine Conservation Analysis (BCMCA) has publicly available spatial data on wave and tidal energy areas of interest in a 1x1 km grid format. The cells have been weighed by a panel of experts according to their relative importance in interest or promise of wave/tidal energy development. This data was used because it incorporates political feasibility when determining relative importance, which would be challenging to account for with the InVEST model.

**Benefits from Commercial Fisheries**

The commercial fisheries model was created in the same manner as the recreational model, using the gridded seascape. The inputs consist of various commercial fisheries spatial files provided by the Fisheries and Oceans Canada (DFO), the Province of BC, and Parks Canada. They were separated based on demersal or pelagic fisheries. Though each grid cell is 1 by 1 km, the actual resolution is coarser, at 4-10 km grids depending upon the fishery.

Human Impacts

Spatial extent of human impacts was modeled using data from the Ban et al. (2010). However, to keep impact and ecosystem service data consistent, the recreational fishery data was not used, as we had already obtained our own for the recreation ecosystem service model. We also acquired our own shipping data from BCMCA that gave a more complete province-wide map of shipping activity on a 200x200m cell grid. This data counted the number of types of ship in a cell (the actual data on shipping intensity per cell was not publicly available though a map of this data was), but we found this to be a suitable proxy of shipping intensity as the resulting map largely mirrored the shipping intensity map. Commercial fisheries were separated based on Ban et al. (2010) classification of demersal destructive, demersal non-destructive low by-catch, pelagic high by-catch, and pelagic low by-catch. These were then combined into 4 rasters. Regional climate impact data from Halpern et al (2008) consisting of 1 by 1 km cells were used to model aragonite saturation state, sea surface temperature change, and UVb change. The other impacts were provided in vector format and converted to 500m rasters. A description of data files is shown in Table S3.

**Calculating Risk from Drivers**

Contrary to Teck et al (2010), who calculated ecosystem vulnerability to individual activities as a linear combination of exposure and consequence, we chose to model ecosystem service risk (*μ_i,j_*) according to a technical model of risk:

$$\mu_{i,j}=P_{i,j}\times C_{i,j}$$

where *P_i,j_* is the exposure of ecosystem service *i*  to an individual occurrence of activity or stressor *j* and *C_i,j_* is the consequence of an individual occurrence of activity or stressor *j* on ecosystem service *i*. This approach allows for the risk to an ecosystem service to be zero if the corresponding *C_i,j_* is zero. We treated *P_i,j_* and *C_i,j_* as linear combinations of exposure and consequence variables, including both ecosystem service supply, service and value dimensions of consequence. The mean expert derived risk scores were used in the cumulative impact model.

**Generating Weights for Risk Criteria**

*P_i,j_* and *C_i,j_* depend on the weight for risk criteria assessed through expert judgement. We calculate *P_i,j_* and *C_i,j_* as

$$P_{i,j}= \sum_{k=1}^{3} W_{k}\times p_{i,j,k}$$

and

$$C_{i,j}= \sum_{l=1}^{4} W_{l}\times c_{i,j,l}$$

where *W_k_* and *W_l_* are the weights of risk criteria *k* and *l* (where $\sum_{k=1}^{3} W_{k}=1$ and $\sum_{l=1}^{4} W_{l}=1$), *p_i,j,k_* is the risk value of activity or stressor *i* on criterion *k* for ecosystem service *j*, and *c_i,j,k_* is the risk value of activity or stressor *i* on criterion *k* for ecosystem service *j*. Similar to previous mapping efforts, we assumed that the weights are similar for all combinations of *i* and *j*, allowing for a single model to be applied to all ecosystem services, in turn allowing for direct comparison between them ([Halpern et al., 2009](#_ENREF_22); [Teck et al., 2010](#_ENREF_50)). To assess the suitability of considering only supply dimensions in cumulative impacts to ecosystem services, the consequence (*C_i,j_*) scores were also calculated with only the supply dimensions included.

To determine risk criteria weights (*W_k_* and *W_l_*), we asked experts to complete discrete choice exercises using a ‘revealed importance’ method ([Neslo, 2011](#_ENREF_37)). This is because it is often unreliable to assess relative criterion importance directly through statements (e.g. asking, “how important is criterion x to variable y”), as this does not force experts to consider multiple criteria simultaneously. We thus asked experts to rank hypothetical scenarios of anthropogenic impacts with plausible values for criteria, split between two ranking exercises. For example, experts might have to rank scenarios of greatest impact where the first scenario has high area of influence scale, low frequency, and low recovery, the second has low area of influence, frequency and recovery time, and the third has a high area of influence, frequency, and recovery time. The first ranking exercise focused on the exposure criteria, and the second focused on the consequence criteria. Splitting the criteria this way allowed us to isolate the importance of criteria within exposure and consequence while limiting the possibility for cognitive overload. Here, we are referring to cognitive research suggesting that people can only receive and process between 3 and 9 variables at once, with recent research emphasizing the lower end of this spectrum ([Gross, 2012](#_ENREF_18); [Miller, 1956](#_ENREF_34)). Asking experts to judge scenarios with 3-4 criteria therefore seemed a more reliable technique than asking them to judge scenarios with 7 criteria.

Resulting ranking data was produced using probabilistic inversion to generate weights. When faced with so-called “inverse problems” where observational performance data is known and information on the structure that generates this data is sought, probabilistic inversion determines a joint distribution of criteria weights that models the distribution of expert preferences given the observed data ([Neslo, 2011](#_ENREF_37); [Teck et al., 2010](#_ENREF_50)). For the purposes of cumulative impact, we use the mean weights from the distribution that best fit the population of expert preference rankings, such that large weights reflect criteria that contribute to consistently high ranking. The same mean weights were used in impact maps that only consider ecosystem service supply dimensions to allow for direct comparison with maps including service and value dimensions. The method produces a joint distribution of weights across the distribution of expert preferences. Therefore, we report on the mean and standard deviation of each criterion to visualize the spread of relative importance among experts, especially the relative importance of ecosystem service supply versus service and value dimensions. Probabilistic inversion generates scenarios by selecting from the probability distribution of exposure and consequence criteria ranks (based on expert the distribution of expert responses), which allows for thousands of nonexclusive combinations. We report the standard deviation rather than the standard error of the mean because we are interested in the distribution among experts, and because probabilistic inversion uses scenario modeling with thousands of scenarios to generate joint distributions, rendering standard error estimates near zero ([White et al., 2014](#_ENREF_51)). We used 20 000 scenarios to generate modeled joint distributions for exposure criteria, and 40 000 scenarios for consequence criteria. Probabilistic inversion was completed using the program UNIVERSE ([Neslo, 2011](#_ENREF_37)).

**Cumulative Impact Model**

After all ecosystem services were modeled, their spatial overlap with all activity and stressors was mapped at a 500x500m cell resolution. The spatial extent of specific ecosystem services served as the boundary for each overlapped map. All activity and stressor layers have associated data related to their level of activity intensity (e.g. density of ships). In order to make the disparate intensity measures comparable, and not influenced by highly skewed intensity data, all intensity scores were log transformed and normalized according to the largest intensity value in each activity and stressor dataset to generate a dimensionless 0-1 intensity scale (Halpern et al. 2008).

Cumulative impact *I_c_* was calculated for each pixel according to the established cumulative impact map formula

$$I_{c}=\sum_{i=1}^{n} D_{i}\times E_{j}\times\mu_{i,j}$$

where *D_i_* is the log-transformed and normalized intensity scores for activity or stressor *i*, E_j_ is the presence or absence of ecosystem service *j*, and *μ_i,j_* is the risk of individual occurrences of activity or stressor *i* on ecosystem service *j* (Halpern et al. 2008). This cumulative impact model assumes impacts are independent, non-negative, and additive in nature. This is an acknowledged limitation of the cumulative impact mapping framework, but allows for an estimate of total impact where great uncertainties persist regarding where, when, and under what conditions non-additive impacts occur ([Halpern and Fujita, 2013](#_ENREF_21)). Cumulative impacts were calculated both including and excluding service and value dimensions to examine the contribution of service and value dimensions on ecosystem services.
