# Supplementary material for: Mapping cumulative impacts to coastal ecosystem services in British Columbia
Source: PLoS One. 2020 May 4;15(5):e0220092. doi: 10.1371/journal.pone.0220092 (PMC7197858; doi:10.1371/journal.pone.0220092)
Supplement: S2 File — (DOC) [file pone.0220092.s009.doc]

**Evaluating Human Threats to Coastal Marine Ecosystem Services of British Columbia**

**Project Overview**

This study aims to evaluate and map human activities and the risks they pose to coastal and marine ecosystem services (ES). The term *ecosystem services* refers, broadly, to the ecological processes that contribute to the benefits that people derive from nature. These include *provisioning services*, such as food (e.g. fish), fibre (e.g. timber) and other raw materials extracted from natural systems; *regulating services*, such as climate regulation and flood control; *cultural services* such as the contribution to recreational experiences, aesthetics and cultural heritage; and *supporting services*, which are processes that maintain the production of the other services. This project builds on previous work to map human impacts on marine ecosystems in British Columbia. It also adapts and expands on methods used by researchers at the National Center for Ecological Analysis and Synthesis, and the Natural Capital Project, to use expert opinion in assessing human impacts on marine ecosystems and map ES provision under different management scenarios.

In this study we are interested in the risks and impacts of human activity to coastal and marine ES of British Columbia within the last ten years. Therefore, please think about human activities and impacts in terms of contemporary effects and risks on current ecosystems. There are select near-future impacts to consider, but these are clearly indicated. The survey should take about one hour to complete.

**PART I: BACKGROUND, EXPERIENCE AND TRAINING**

**1. Main affiliation:**

**2. Other affiliations:**

**3. Type of position (please select one of the following, and place the corresponding letter in the box):**

1. academic
2. agency (federal/provincial)
3. non-governmental organization
4. private company

**4. Age:**

**5. Gender:**

A) Male

B) Female

C) Other

**6. Highest degree earned:**

**7. Year of degree:**

Use the following map to orient yourself for the following questions:


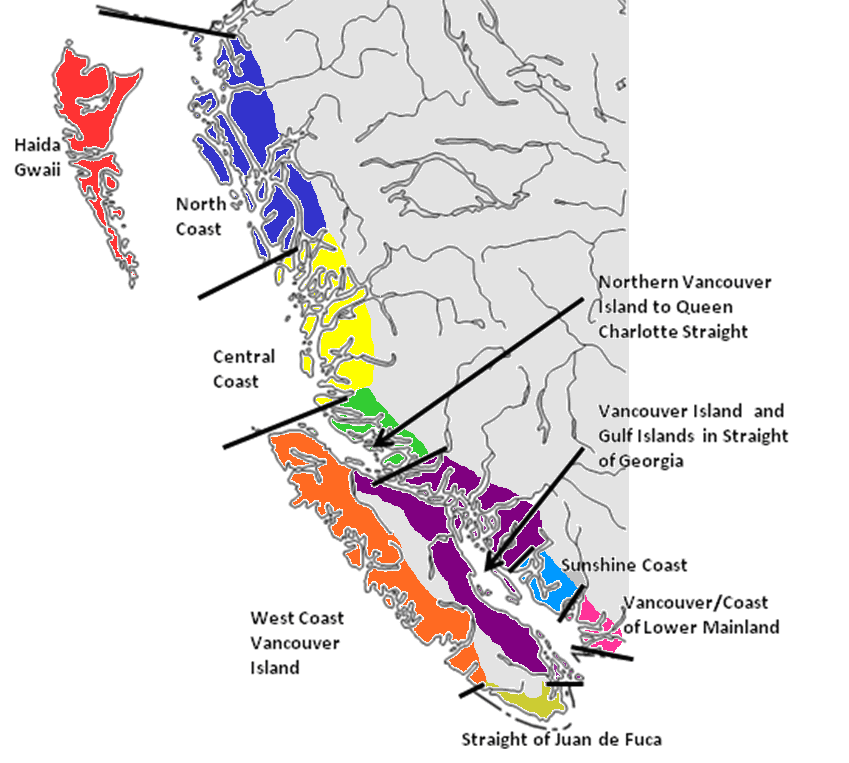


**8. How many years of scientific experience do you have within each geographic region**? *(please indicate the number of years beside each area listed below)*

If your knowledge doesn’t stem from a particular area, write “NA” in the space provided:

| Haida Gwaii |  |
| --- | --- |
| North Coast |  |
| Central Coast |  |
| Northern Vancouver Island to Queen Charlotte Straight |  |
| Vancouver Island and Gulf Islands |  |
| Sunshine Coast |  |
| Vancouver/Coast of Lower Mainland |  |
| West Coast Vancouver Island |  |
| Straight of Juan de Fuca |  |

**9. Which geographic region(s) does your knowledge apply to for this survey?**

Please indicate one or more of the areas listed below by placing an ‘X’ next to the area.

*If indicating multiple regions please let us know whether you think human activities affect* *these areas differently by stating so next to the region’s name, so that we may provide any needed additional forms.*

| Haida Gwaii |  |
| --- | --- |
| North Coast |  |
| Central Coast |  |
| Northern Vancouver Island to Queen Charlotte Straight |  |
| Vancouver Island and Gulf Islands |  |
| Sunshine Coast |  |
| Vancouver/Coast of Lower Mainland |  |
| West Coast Vancouver Island |  |
| Straight of Juan de Fuca |  |

If you feel that your experience with a subset of a region doesn’t apply to the rest of the region, please list the specific location(s) you want to consider:

**10. How many years of experience do you have with each sector related to ecosystem services listed below?**

*Please note the number of years in each space provided, including zero for no experience*:

| Renewable energy potential from wave or wind energy |  |
| --- | --- |
| Commercial aquaculture |  |
| Commercial wild fisheries |  |
| Recreational fisheries |  |
| Recreational/subsistence fisheries |  |
| Coastal protection and erosion |  |
| Landscape planning for aesthetic quality |  |
| Marine recreation |  |
| Marine carbon sequestration |  |
| Water quality |  |
| Habitat (e.g. seagrass, kelp forests) |  |

**11. Given your expertise, please specify the sector you would like to focus on for this survey by placing an ‘X’ next to the relevant sector.**

If you have expertise in multiple sectors, please let us know so that we may provide you the necessary additional forms:

| Renewable energy potential from wave or wind energy |  |
| --- | --- |
| Commercial aquaculture |  |
| Commercial wild fisheries |  |
| Recreational fisheries |  |
| Recreational/subsistence fisheries |  |
| Coastal protection and erosion |  |
| Landscape planning for aesthetic quality |  |
| Marine recreation |  |
| Marine carbon sequestration |  |
| Water quality |  |
| Habitat (e.g. seagrass, kelp forests) |  |

**PART II: RANKING RISK OF ACTIVITIES**

**Risk of ecosystem services (ES) to Human Activities**

As we are interested in understanding the cumulative risks of human activities to coastal ES, we consider risk indices as a function of exposure/probability of ES to an activity and consequence/vulnerability of an ES to an activity, according to the risk equation

Risk = Exposure × Consequence

Drawing on this equation we consider Exposure to be a combination of **recovery time** of an ES to an activity, **frequency** of an activity affecting an ES, and **area of influence** of an activity to an ES. We consider Consequence to include the biophysical components of ES (supply metrics), including the **magnitude of risk to ES through risks to biophysical producers/production (Magnitude of Risk)** and **ecological community extent,** as well as the social components of ES related to the ES production actually used and enjoyed by people (service metrics), including the **quality of service** and **access to a service.**

Each risk-by-ES interaction likely has a specific vulnerability score, given the nature of how ecosystems and people respond to risks. These are the scores we are trying to estimate with this survey. We are interested in the total risk of human activities to ES provision, including all direct and indirect risks. Please keep this in mind when providing scores for a risk on a service. We recognize that some human activities can actually enhance ecosystem services (e.g. building wharfs and ports can enhance the access to fishing sites, aquaculture enhances food production). For the sake of simplicity, however, here we evaluate only the risk of human activities to ES, through the dimensions of ES vulnerability outlined below.

The table on the following page is presented to provide a guide for thinking about the scale of human activities and their impacts when considering their risks to ES. We ask you to consider human activities on the scale of individual events, and to think of the **frequency** criterion as a measure of how often individual events occur.

As ES are necessarily socio-ecological, we identify biophysical and social dimensions of ES vulnerability. Following the Millennium Ecosystem Assessment Condition and Trends Working Group definition of vulnerability, we define the social and biophysical aspects of vulnerability as a function of 1) exposure, 2) sensitivity, and 3) resilience of ES.

Drawing on this definition we consider ES vulnerability to include the biophysical and social components of an impact to ES. We consider the impact to ES production, measured as **magnitude change in ES producers** and **ecological community extent**, and the resilience of ES to a given impact, measured as **recovery time** (these are biophysical considerations of sensitivity), as well as **frequency** and **spatial scale** **of the impact** (exposure), and the **quality of service** and **access to a service** (social considerations of sensitivity).

Each risk-by-ES interaction likely has a specific vulnerability score, given the nature of how ecosystems and people respond to risks. These are the scores we are trying to estimate with this survey. We are interested in the total risk of human activities to ES provision, including all direct and indirect risks. Please keep this in mind when providing scores for a risk on a service. We recognize that some human activities can actually enhance ecosystem services (e.g. building wharfs and ports can enhance the access to fishing sites, aquaculture enhances food production). For the sake of simplicity, however, here we evaluate only the risk of human activities to ES, through the dimensions of ES vulnerability outlined below.

The table on the following page is presented to provide a guide for thinking about the scale of human activities and their impacts when considering their risks to ES. We ask you to consider human activities on the scale of individual events, and to think of the **frequency** criterion as a measure of how often individual events occur.

| **Activity Type** | **Scale of Consideration for ‘Individual Events’** |
| --- | --- |
| **Agriculture** | One farm and its associated risks including silt and pesticide runoff |
| **Commercial fishing** | One fishing vessel of the specific fishing type indicated and its associated risks such as catch, bycatch and lost fishing gear |
| **Finfish aquaculture** | One aquaculture facility and its associated effects such as disease and nutrient transmission |
| **Human settlements** | One human house and associated risks such as pollutants |
| **Industry** | One industrial building (e.g. factory) including associated risks such as pollutants |
| **Large boat traffic** | One large boat or ship and associated risks such as strikes/collisions, acoustic impacts, and illegal dumping of oily wastes and greywater |
| **Log dumping, handling, storage** | One log-dumping site and associated risks such as scouring and leachates |
| **Ocean dumping** | One dump event in a site designated for ocean dumping of nontoxic materials |
| **Onshore mining** | One mining pit, and associated risks from discharge and drainage |
| **Ports, marinas and harbors** | One marina/port/harbor, including the dredging needed to create it and associated risks of contaminants and breakwaters |
| **Small docks, ramps, wharves** | One dock/ramp/wharf, and associated risks such as from shading and contaminants |
| **Pulp and paper** | One pulp mill and associated risks including toxic effluent and leaks |
| **Recreational fishing** | One fishing vessel of the specific fishing type indicated and its associated risks such as catch, bycatch and lost fishing gear |
| **Shellfish aquaculture** | One aquaculture facility including its risks to intertidal habitats and risks of invasive species release |
| **Climate change** | As a global change stressor, consider an event to cover the entire phenomenon of climate change |
| **Offshore drilling** | One oil rig platform, including risks from construction and oil spills |

**Vulnerability Criteria**

***Biophysical Dimensions***

The biophysical dimensions of ES account for the species and ecosystem components that contribute to ES production. They include:

**Recovery time** - *the average time (in years) required for the affected ES to return to its former level of provision, following disturbance by a particular activity*.

Fractions represent times shorter than a single year. Recovery of the ES is related to the resilience of the system to a type of risk. It relates to recovery of a system within a site as well as the necessary species or environmental components that make up the biophysical producers/production of an ES from surrounding areas to recolonize a site. "Biophysical producers" indicates the specific species responsible for producing the ecosystem service under consideration.

**Magnitude of risk to ES through risks to biophysical producers/production (Magnitude of Risk)** - *the degree to which an ES is at risk due to potential negative impacts of an activity on the ES’s biophysical producers/production, on a 0 to 1 scale.*

For renewable energy consider the risk to potential renewable energy generated by wind or waves; for fishery-related ES consider the risk to fishery yield; consider the change in the amount of carbon that can be sequestered for marine carbon; consider the change in erosion risk for coastal protection. Magnitude of risk to ES affected by an activity also addresses the diversity of ES producers responsible for ES. If a risk affects a high proportion of ES producers, that ES is likely more vulnerable and less resistant to the risk.

**Community extent** – *the extent of risk on the underlying ecological community responsible for producing an ecosystem service.*

Human activities can affect the primary biophysical producers of ecosystem services directly or indirectly through associated species and habitats. More extensive risks may also affect primary biophysical producers of ecosystem services *and* associated species and habitats. If you perceive no ecological community components to the ES you are addressing, choose ‘0’ (no risk) using the scale. Community impact is scored on the following scale:

0: An activity might occur on some part of the ecosystem, but does not affect the biophysical producer of the ecosystem service.

1: The biophysical producers of the ecosystem service (through direct or indirect impact)

2: The biophysical producers plus supporting species

3: The biophysical producers plus supporting species plus surrounding habitat structure

"Biophysical producers plus supporting species" indicates that an activity has broader impacts across the biological community without affecting the underlying habitat. For example, shipping would score a 1 for marine recreation if considering that ship strikes impact a sought-after species of whale. Similarly, fishing would score a 2 for contribution to the whale-watching industry if marine mammal abundance and distribution are at risk indirectly through a decreased prey base. Larger phenomena such as climate change could rank as a 3 for provision of rockfish fishing if kelp forests are expected to decline in extent, given that kelp provide biogenic habitat for rockfish and many supporting species.

**Area of Influence -** *the spatial influence of a single event of an activity on the area where a service is provided, where a risk may be direct or indirect, measured in km^2^.*

This is a measure of the exposure of an ecosystem service to a human activity. It represents the impact of a *single* event of an activity, not the aggregate or cumulative presence of the activity across the seascape. Trawling, in total, may impact thousands of square kilometres, but a single trawling event may cover less than 1 to 10 km^2^. It is this second number we are interested in. If onshore mining negatively impacts the view of an entire bay, then the spatial influence of onshore mining to aesthetics is the entire bay.

**Frequency** – *the average annual frequency (days per year) of individual events of an activity at a particular location*.

It is important to remember that frequency is not a measure of duration, but how many times an activity occurs in an area in a year. For example, if fishing for groundfish occurs everywhere in a region, but on average only occurs at any given location 3 times a year, the frequency would be 3 per year. Duration will be captured by the "recovery time" dimension. In cases where an activity is ongoing (i.e. there is no pinpoint "event") the frequency of that activity should be counted every day (i.e. 365). Fractions represent return-times longer than a year (e.g. 1/10 = once a decade).

***Social Dimensions***

The social aspects of ecosystem services include dimensions of both supply and demand for ecosystem services, including access to areas, and enjoyment that people derive from that access. We do not address other aspects of demand (e.g., global market trends in ecotourism), which vary with factors beyond an individual natural system. Rather, we strive to capture only the aspects that change as a result of changes to the ecosystem.

**Access to a Service** - *the change in the ability and rights of people to access an area so they can benefit from the ecosystem service in question, measured on a 0 to 1 scale.*

A 0 correspond to zero or positive effect, whereas a "1" indicates a complete loss of access. If log dumping and handling restricts access to subsistence fishing in an area by reducing the area that can be fished by 50%, then this would be scored as 0.5. If it completely closes an area that people use to fish, then this would score as 1.

**Quality of Service** - *the change in the enjoyment or benefit that people procure from an ecosystem service given the same quantity of good or experience, measured on a 0 to 1 scale.*

Assuming that there are no limitations for someone to access a service, evaluate how an activity would affect the enjoyment of that service. This is intended to capture the risk of human activities to the intangible benefits that people derive from ES. For each service you are filling out a survey for, consider this dimension in the context of that service. A 0 indicates no (or even positive) risk and a 1 refers to a complete loss of enjoyment associated with service provision. For landscape aesthetics and recreation, consider the change in the quality of the scenery or enjoyment; consider the change in the constituents of water quality (dissolved oxygen, pathogens, nutrients, etc.) that affect enjoyment of shellfish (potentially through human health risk). If agricultural runoff reduces the water quality of an area to the point where shellfish harvest poses a major health hazard, this would be scored as a 1.

**RANKING ACTIVITIES USING OUR PROVIDED VULNERABILITY MEASURES:**

Use the following table as a reference for the scales used for the vulnerability criteria

| **Vulnerability Criteria** | **Scale** |
| --- | --- |
| **Quality of Service** - *the change in the enjoyment or benefit that people procure from an ecosystem service given the same quantity of good or experience* | 0-1 scale |
| **Access to Service** - *the change in the ability and rights of people to access an area so they can benefit from the ecosystem service in question.* | 0-1 scale |
| **Recovery Time** - *the average time required for the affected ES to return to its former level of provision, following disturbance by a particular activity*. | Years |
| **Magnitude of Risk** - *the degree to which an ES is at risk due to potential negative impacts of an activity on the ES’s biophysical producers/production* | 0 - 1 scale |
| **Community Extent** - *the extent of risk on the underlying ecological community responsible for producing an ecosystem service* | 0, 1, 2, or 3 |
| **Area of Influence** - *the spatial influence of a single event of an activity on the area where a service is provided, where a risk may be direct or indirect* | km^2^ |
| **Frequency** - *the average annual frequency (days per year) of individual events of an activity at a particular location* | days/year |

Below, please rank the activities by their severity of risk based upon the vulnerability measures provided. There are 6 scenario boxes, each with 7 scenarios. We ask you to determine and rank the scenarios from the greatest risk to the least(1-7), with 1 indicating the greatest risk and 7 the least. The rankings will be used to create a predictive model for assessing the overall ecological risks of human activities when vulnerability measures are known. We will give you the opportunity to quantify the vulnerability measures for your region(s) and ecosystem services(s) of expertise in Part III of the survey.

Box 1

| **Scenario** | **Quality of Service** | **Access to Service** | **Recovery Time** | **Magnitude of Risk** | **Community Extent** | **Area of Influence** | **Frequency** | **Rank** |
| --- | --- | --- | --- | --- | --- | --- | --- | --- |
| A | 0.3 | 0.4 | 5 | 0.75 | 3 | 500 | 0 |  |
| B | 0.05 | 0.55 | 2 | 0.1 | 1 | 10 | 10 |  |
| C | 0.5 | 0 | 3 | 0.4 | 2 | 100 | 1 |  |
| D | 0.75 | 0.15 | 25 | 0.85 | 3 | 50000 | 180 |  |
| E | 0.2 | 0.7 | 1 | 0.2 | 1 | 1 | 300 |  |
| F | 0.35 | 0.25 | 1 | 0.65 | 2 | 2 | 150 |  |
| G | 0.65 | 0.95 | 20 | 0.9 | 3 | 30000 | 1/2 |  |

Box 2

| **Scenario** | **Quality of Service** | **Access to Service** | **Recovery Time** | **Magnitude of Risk** | **Community Extent** | **Area of Influence** | **Frequency** | **Rank** |
| --- | --- | --- | --- | --- | --- | --- | --- | --- |
| A | 0.8 | 0.1 | 5 | 0.65 | 2 | 10 | 0 |  |
| B | 0.85 | 0.1 | 1 | 0.8 | 2 | 50 | 1 |  |
| C | 0.45 | 0.25 | 0.5 | 0.15 | 1 | 3 | 10 |  |
| D | 0.3 | 0.4 | 5 | 0.75 | 3 | 500 | 0 |  |
| E | 0.6 | 0.75 | 5 | 0.35 | 1 | 5 | 0 |  |
| F | 0.5 | 0 | 3 | 0.4 | 2 | 100 | 1 |  |
| G | 0.25 | 0.05 | 1 | 0.55 | 1 | 5 | 5 |  |

Box 3

| **Scenario** | **Quality of Service** | **Access to Service** | **Recovery Time** | **Magnitude of Risk** | **Community Extent** | **Area of Influence** | **Frequency** | **Rank** |
| --- | --- | --- | --- | --- | --- | --- | --- | --- |
| A | 0 | 0.15 | 3 | 0.35 | 2 | 10 | 50 |  |
| B | 0.4 | 0.7 | 5 | 0.5 | 2 | 15 | 200 |  |
| C | 0.85 | 0.1 | 1 | 0.8 | 2 | 50 | 1 |  |
| D | 0.15 | 0.2 | 5 | 0.2 | 1 | 10 | 1/2 |  |
| E | 0.6 | 0.6 | 5 | 0.8 | 3 | 5 | 0 |  |
| F | 0.45 | 0.45 | 1 | 0.25 | 1 | 100 | 1 |  |
| G | 0.45 | 0.25 | 0.5 | 0.15 | 1 | 3 | 10 |  |

Box 4

| **Scenario** | **Quality of Service** | **Access to Service** | **Recovery Time** | **Magnitude of Risk** | **Community Extent** | **Area of Influence** | **Frequency** | **Rank** |
| --- | --- | --- | --- | --- | --- | --- | --- | --- |
| A | 1 | 0.6 | 10 | 0.75 | 3 | 1000 | 20 |  |
| B | 0.15 | 0.2 | 5 | 0.2 | 1 | 10 | 1/2 |  |
| C | 0.95 | 1 | 10 | 0.6 | 2 | 150 | 250 |  |
| D | 0.35 | 0.9 | 5 | 0.25 | 1 | 50 | 0 |  |
| E | 0.5 | 0.85 | 25 | 0.8 | 3 | 40000 | 360 |  |
| F | 0 | 0.15 | 3 | 0.35 | 2 | 10 | 50 |  |
| G | 0.75 | 0.5 | 70 | 0.9 | 2 | 1000 | 100 |  |

Box 5

| **Scenario** | **Quality of Service** | **Access to Service** | **Recovery Time** | **Magnitude of Risk** | **Community Extent** | **Area of Influence** | **Frequency** | **Rank** |
| --- | --- | --- | --- | --- | --- | --- | --- | --- |
| A | 0.2 | 1 | 0.1 | 0.25 | 3 | 0.5 | 1/2 |  |
| B | 0.75 | 0.5 | 70 | 0.9 | 2 | 1000 | 100 |  |
| C | 0.05 | 0.8 | 25 | 0.15 | 2 | 100 | 300 |  |
| D | 1 | 0.8 | 20 | 0.5 | 2 | 250 | 0 |  |
| E | 0.25 | 0.4 | 5 | 0.15 | 1 | 10 | 1/2 |  |
| F | 0.35 | 0.9 | 5 | 0.25 | 1 | 50 | 0 |  |
| G | 0.85 | 0.2 | 1 | 0.3 | 1 | 15 | 50 |  |

Box 6

| **Scenario** | **Quality of Service** | **Access to Service** | **Recovery Time** | **Magnitude of Risk** | **Community Extent** | **Area of Influence** | **Frequency** | **Rank** |
| --- | --- | --- | --- | --- | --- | --- | --- | --- |
| A | 1 | 0.8 | 20 | 0.5 | 2 | 250 | 0 |  |
| B | 0.4 | 0.75 | 5 | 0.7 | 3 | 50 | 200 |  |
| C | 0.15 | 0.85 | 20 | 0.4 | 2 | 500 | 0 |  |
| D | 0.25 | 0.4 | 5 | 0.15 | 1 | 10 | 1/2 |  |
| E | 0.1 | 0.55 | 5 | 0.3 | 1 | 10 | 150 |  |
| F | 0.4 | 0.3 | 2 | 0.35 | 2 | 5 | 75 |  |
| G | 0.6 | 0.2 | 15 | 0.45 | 3 | 50 | 1/5 |  |

**PART III. VULNERABILITY MEASURES**

Please use the same approach to evaluating vulnerability outlined in Part II; the vulnerability scores capture the nature of an activity where it exists. We also include some potential near-future human activities (within the next decade) that could pose important risks to ES. Drawing upon your experience in the region(s) where you work, we ask you to:

**1.** Indicate whether the human activity has a direct ("D") or indirect ("I") impact on the ecosystem service you are evaluating in the left column. If you think an activity affects an ES directly and indirectly, enter both "D" and "I". If it doesn't affect the ecosystem service, write 0.

**2.**Give your best estimate, as well as perceived upper and lower estimates for the vulnerability measures ("LB" = lower bound, "best" = best estimate, "UB" = upper bound), using the same scales you used to rank scenarios above.

**3.** If you are unable to assess a particular indicator (due to data gaps, lack of experience or uncertainty) please write “don’t know” in the blank space.

**4.** If an activity does not affect your ecosystem, please consider all scores to be “0.”

In all cases, please use your best judgment, drawing on published and unpublished empirical data, experiments, reviews, and personal experience in the field to assess vulnerability of your study ecosystem service in your chosen region. One way to approach this table is to consider your service and work your way across the row for each human activity, considering each vulnerability criterion in turn. Alternatively, you can focus on a single vulnerability criterion at a time, making sure you fully understand it, and work down the column of that criterion for each human activity, then repeat for every criterion.

| **Activity** | **Direct/ Indirect**  **(D/ I)** | **Quality of service affected** | | | | **Access to Service** | | | **Recovery Time** | | | | | **Magnitude of Risk** | | | **Community Extent** | | | | **Area of Influence** | | | | | **Frequency** | | | | |
| --- | --- | --- | --- | --- | --- | --- | --- | --- | --- | --- | --- | --- | --- | --- | --- | --- | --- | --- | --- | --- | --- | --- | --- | --- | --- | --- | --- | --- | --- | --- |
|  |  | LB | Best | | UB | LB | Best | UB | LB | | Best | | UB | LB | Best | UB | LB | | Best | UB | LB | | Best | UB | | LB | | Best | UB | |
| Agriculture |  |  |  |  | |  |  |  |  |  | |  | |  |  |  |  |  | |  |  |  | | |  |  |  | | |  |
| Commercial fishing: Bottom trawling |  |  |  |  | |  |  |  |  |  | |  | |  |  |  |  |  | |  |  |  | | |  |  |  | | |  |
| Commercial fishing: Crab |  |  |  |  | |  |  |  |  |  | |  | |  |  |  |  |  | |  |  |  | | |  |  |  | | |  |
| Commercial fishing: Geoduck |  |  |  |  | |  |  |  |  |  | |  | |  |  |  |  |  | |  |  |  | | |  |  |  | | |  |
| Commercial fishing: Gooseneck barnacle |  |  |  |  | |  |  |  |  |  | |  | |  |  |  |  |  | |  |  |  | | |  |  |  | | |  |
| Commercial fishing: Green urchin |  |  |  |  | |  |  |  |  |  | |  | |  |  |  |  |  | |  |  |  | | |  |  |  | | |  |
| Commercial fishing: Groundfish ZN |  |  |  |  | |  |  |  |  |  | |  | |  |  |  |  |  | |  |  |  | | |  |  |  | | |  |
| Commercial fishing: Herring |  |  |  |  | |  |  |  |  |  | |  | |  |  |  |  |  | |  |  |  | | |  |  |  | | |  |
| Commercial fishing: Herring roe |  |  |  |  | |  |  |  |  |  | |  | |  |  |  |  |  | |  |  |  | | |  |  |  | | |  |
| Commercial fishing: Krill |  |  |  |  | |  |  |  |  |  | |  | |  |  |  |  |  | |  |  |  | | |  |  |  | | |  |

| **Activity** | **Direct/ Indirect**  **(D/ I)** | **Quality of service affected** | | | **Access to Service** | | | **Recovery Time** | | | | | **Magnitude of Risk** | | | **Community Extent** | | | | **Area of Influence** | | | | | | **Frequency** | | | | |
| --- | --- | --- | --- | --- | --- | --- | --- | --- | --- | --- | --- | --- | --- | --- | --- | --- | --- | --- | --- | --- | --- | --- | --- | --- | --- | --- | --- | --- | --- | --- |
|  |  | LB | Best | UB | LB | Best | UB | LB | | Best | | UB | LB | Best | UB | LB | | Best | UB | LB | | Best | | UB | | LB | | Best | UB | |
| Commercial fishing: Octopus |  |  |  |  |  |  |  |  |  | |  | |  |  |  |  |  | |  |  | | |  | |  |  |  | | |  |
| Commercial fishing: Prawn trap |  |  |  |  |  |  |  |  |  | |  | |  |  |  |  |  | |  |  | | |  | |  |  |  | | |  |
| Commercial fishing: Red urchin |  |  |  |  |  |  |  |  |  | |  | |  |  |  |  |  | |  |  | | |  | |  |  |  | | |  |
| Commercial fishing: Sablefish longline |  |  |  |  |  |  |  |  |  | |  | |  |  |  |  |  | |  |  | | |  | |  |  |  | | |  |
| Commercial fishing: Sablefish trap |  |  |  |  |  |  |  |  |  | |  | |  |  |  |  |  | |  |  | | |  | |  |  |  | | |  |
| Commercial fishing: Salmon net |  |  |  |  |  |  |  |  |  | |  | |  |  |  |  |  | |  |  | | |  | |  |  |  | | |  |
| Commercial fishing: Salmon troll |  |  |  |  |  |  |  |  |  | |  | |  |  |  |  |  | |  |  | | |  | |  |  |  | | |  |
| Commercial fishing: Scallop |  |  |  |  |  |  |  |  |  | |  | |  |  |  |  |  | |  |  |  | | | |  |  |  | | |  |
| Commercial fishing: Schedule II |  |  |  |  |  |  |  |  |  | |  | |  |  |  |  |  | |  |  |  | | | |  |  |  | | |  |
| Commercial fishing: Sea cucumber |  |  |  |  |  |  |  |  |  | |  | |  |  |  |  |  | |  |  |  | | | |  |  |  | | |  |

| **Activity** | **Direct/ Indirect**  **(D/ I)** | **Quality of service affected** | | | | **Access to Service** | | | **Recovery Time** | | | | | **Magnitude of Risk** | | | **Community Extent** | | | | **Area of Influence** | | | | | **Frequency** | | | | |
| --- | --- | --- | --- | --- | --- | --- | --- | --- | --- | --- | --- | --- | --- | --- | --- | --- | --- | --- | --- | --- | --- | --- | --- | --- | --- | --- | --- | --- | --- | --- |
|  |  | LB | Best | | UB | LB | Best | UB | LB | | Best | | UB | LB | Best | UB | LB | | Best | UB | LB | | Best | UB | | LB | | Best | UB | |
| Commercial fishing: Shrimp trawl |  |  |  |  | |  |  |  |  |  | |  | |  |  |  |  |  | |  |  |  | | |  |  |  | | |  |
| Commercial fishing: Squid |  |  |  |  | |  |  |  |  |  | |  | |  |  |  |  |  | |  |  |  | | |  |  |  | | |  |
| Finfish aquaculture |  |  |  |  | |  |  |  |  |  | |  | |  |  |  |  |  | |  |  |  | | |  |  |  | | |  |
| Human settlements |  |  |  |  | |  |  |  |  |  | |  | |  |  |  |  |  | |  |  |  | | |  |  |  | | |  |
| Industry |  |  |  |  | |  |  |  |  |  | |  | |  |  |  |  |  | |  |  |  | | |  |  |  | | |  |
| Large boat traffic (tankers, cruise ships, etc.) |  |  |  |  | |  |  |  |  |  | |  | |  |  |  |  |  | |  |  |  | | |  |  |  | | |  |
| Log dumping, handling and storage |  |  |  |  | |  |  |  |  |  | |  | |  |  |  |  |  | |  |  |  | | |  |  |  | | |  |
| Ocean dumping (designated sites for non-toxic materials) |  |  |  |  | |  |  |  |  |  | |  | |  |  |  |  |  | |  |  |  | | |  |  |  | | |  |

| **Activity** | **Direct/ Indirect**  **(D/ I)** | **Quality of service affected** | | | | **Access to Service** | | | **Recovery Time** | | | | | **Magnitude of Risk** | | | **Community Extent** | | | | **Area of Influence** | | | | | **Frequency** | | | | |
| --- | --- | --- | --- | --- | --- | --- | --- | --- | --- | --- | --- | --- | --- | --- | --- | --- | --- | --- | --- | --- | --- | --- | --- | --- | --- | --- | --- | --- | --- | --- |
|  |  | LB | Best | | UB | LB | Best | UB | LB | | Best | | UB | LB | Best | UB | LB | | Best | UB | LB | | Best | UB | | LB | | Best | UB | |
| Onshore mining |  |  |  |  | |  |  |  |  |  | |  | |  |  |  |  |  | |  |  |  | | |  |  |  | | |  |
| Ports, Marinas and harbors |  |  |  |  | |  |  |  |  |  | |  | |  |  |  |  |  | |  |  |  | | |  |  |  | | |  |
| Pulp and paper |  |  |  |  | |  |  |  |  |  | |  | |  |  |  |  |  | |  |  |  | | |  |  |  | | |  |
| Recreational fishing: Dive |  |  |  |  | |  |  |  |  |  | |  | |  |  |  |  |  | |  |  |  | | |  |  |  | | |  |
| Recreational fishing: Line |  |  |  |  | |  |  |  |  |  | |  | |  |  |  |  |  | |  |  |  | | |  |  |  | | |  |
| Recreational fishing: Lodges |  |  |  |  | |  |  |  |  |  | |  | |  |  |  |  |  | |  |  |  | | |  |  |  | | |  |
| Recreational fishing: Trap |  |  |  |  | |  |  |  |  |  | |  | |  |  |  |  |  | |  |  |  | | |  |  |  | | |  |
| Recreational fishing: Unspecified |  |  |  |  | |  |  |  |  |  | |  | |  |  |  |  |  | |  |  |  | | |  |  |  | | |  |
| Shellfish aquaculture |  |  |  |  | |  |  |  |  |  | |  | |  |  |  |  |  | |  |  |  | | |  |  |  | | |  |
| Invasive Species |  |  |  |  | |  |  |  |  |  | |  | |  |  |  |  |  | |  |  |  | | |  |  |  | | |  |

| **Activity** | **Direct/ Indirect**  **(D/ I)** | **Quality of service affected** | | | | **Access to Service** | | | **Recovery Time** | | | | | **Magnitude of Risk** | | | **Community Extent** | | | | **Area of Influence** | | | | | **Frequency** | | | | |
| --- | --- | --- | --- | --- | --- | --- | --- | --- | --- | --- | --- | --- | --- | --- | --- | --- | --- | --- | --- | --- | --- | --- | --- | --- | --- | --- | --- | --- | --- | --- |
|  |  | LB | Best | | UB | LB | Best | UB | LB | | Best | | UB | LB | Best | UB | LB | | Best | UB | LB | | Best | UB | | LB | | Best | UB | |
| Small docks, ramps, wharves |  |  |  |  | |  |  |  |  |  | |  | |  |  |  |  |  | |  |  |  | | |  |  |  | | |  |
| Climate change: ocean acidification |  |  |  |  | |  |  |  |  |  | |  | |  |  |  |  |  | |  |  |  | | |  |  |  | | |  |
| Climate change: sea level rise |  |  |  |  | |  |  |  |  |  | |  | |  |  |  |  |  | |  |  |  | | |  |  |  | | |  |
| Climate change: sea temp. change |  |  |  |  | |  |  |  |  |  | |  | |  |  |  |  |  | |  |  |  | | |  |  |  | | |  |
| Climate change: UV change |  |  |  |  | |  |  |  |  |  | |  | |  |  |  |  |  | |  |  |  | | |  |  |  | | |  |
| Future Risk: Large boat traffic assuming Northern Gateway Pipeline |  |  |  |  | |  |  |  |  |  | |  | |  |  |  |  |  | |  |  |  | | |  |  |  | | |  |
| Future Risk: offshore drilling |  |  |  |  | |  |  |  |  |  | |  | |  |  |  |  |  | |  |  |  | | |  |  |  | | |  |

**PART IV. SUPPLEMENTARY INFORMATION**

**1. If you were to refine the criteria we used to assess and measure ES vulnerability, how would you? This could include criteria that you would add, omit or revise.**

**2. If you indicated that any of the human activities impact the focal ES of this survey in an indirect fashion, how would you characterize the pathway of the impact? Use the space provided to draw out/explain the indirect path of the impact for as many impacts as you would like.**

**3. If you think of human activities that have not been included in this survey that you believe will pose important risks to coastal ES production in the next decade, please list them below, as well as the relevant ES that will be affected.**

**4. Comments or questions?**
