# Supplementary material for: Mapping cumulative impacts to coastal ecosystem services in British Columbia
Source: PLoS One. 2020 May 4;15(5):e0220092. doi: 10.1371/journal.pone.0220092 (PMC7197858; doi:10.1371/journal.pone.0220092)
Supplement: Attachment — Submitted filename: response to reviews Dec5_update.docx [file pone.0220092.s011.docx]

**We thank the editor and the two reviewer’s for their thoughtful feedback. We believe that the manuscript is now much stronger as a result of addressing the comments and helpful suggestions. Below please find our responses to the editor and reviewer comments in bold red.**

Journal Requirements:

When submitting your revision, we need you to address these additional
requirements.

Please ensure that your manuscript meets PLOS ONE's style requirements,
including those for file naming. The PLOS ONE style templates can be
found at

<http://www.journals.plos.org/plosone/s/file?id=wjVg/PLOSOne_formatting_sample_main_body.pdf>
and
<http://www.journals.plos.org/plosone/s/file?id=ba62/PLOSOne_formatting_sample_title_authors_affiliations.pdf>

**We have gone through the manuscript and ensured that it meets PLOS ONE’s style requirements.**

1. Please include additional information regarding the survey or
questionnaire used in the study and ensure that you have provided
sufficient details that others could replicate the analyses. For
instance, if you developed a questionnaire as part of this study and it
is not under a copyright more restrictive than CC-BY, please include a
copy as Supporting Information.

**We have now added a paper copy of the survey as a supplement, though the survey was actually conducted online. We have also ensured that the methods of the manuscript describe the kind of information collected in the survey.**

2. We note that you have stated that you will provide repository
information for your data at acceptance. Should your manuscript be
accepted for publication, we will hold it until you provide the relevant
accession numbers or DOIs necessary to access your data. If you wish to
make changes to your Data Availability statement, please describe these
changes in your cover letter and we will update your Data Availability
statement to reflect the information you provide.

**We still intend to submit the data to a public repository.**

[Note: HTML markup is below. Please do not edit.]

Reviewers' comments:

Reviewer's Responses to Questions

COMMENTS TO THE AUTHOR

1. Is the manuscript technically sound, and do the data support the
conclusions?

The manuscript must describe a technically sound piece of scientific
research with data that supports the conclusions. Experiments must have
been conducted rigorously, with appropriate controls, replication, and
sample sizes. The conclusions must be drawn appropriately based on the
data presented.

Reviewer #1: Yes

Reviewer #2: No

-------------------------

2. Has the statistical analysis been performed appropriately and
rigorously?

Reviewer #1: Yes

Reviewer #2: No

-------------------------

3. Have the authors made all data underlying the findings in their
manuscript fully available?

The PLOS Data policy [2] requires authors to make all data underlying
the findings described in their manuscript fully available without
restriction, with rare exception (please refer to the Data Availability
Statement in the manuscript PDF file). The data should be provided as
part of the manuscript or its supporting information, or deposited to a
public repository. For example, in addition to summary statistics, the
data points behind means, medians and variance measures should be
available. If there are restrictions on publicly sharing data—e.g.
participant privacy or use of data from a third party—those must be
specified.

Reviewer #1: No

Reviewer #2: No

-------------------------

4. Is the manuscript presented in an intelligible fashion and written in
standard English?

PLOS ONE does not copyedit accepted manuscripts, so the language in
submitted articles must be clear, correct, and unambiguous. Any
typographical or grammatical errors should be corrected at revision, so
please note any specific errors here.

Reviewer #1: Yes

Reviewer #2: No

-------------------------

5. Review Comments to the Author

Please use the space provided to explain your answers to the questions
above. You may also include additional comments for the author,
including concerns about dual publication, research ethics, or
publication ethics. (Please upload your review as an attachment if it
exceeds 20,000 characters)

Reviewer #1: The study presented here provides a novel method for
estimating the human activities impact on ecosystem services. This is a
very interesting and important field of research, among others because
it provides valuable knowledge that could be applied to informed
management and decision making. From the scientific point of view, the
linkages between human activities, the pressures they produce and the
associated environmental impact and their consequences in ecosystem
services provisioning, is still a very complex topic and not well-known.
Adopting the British Columbia as case study, the authors describe a
comprehensive process conducted for the obtention of the input data,
model development and implementation. In one hand, the authors have
estimated the ecosystem services production and their spatial
distribution by using real data or using already available and broadly
used models (InVEST) models for different ecosystem services). On the
other hand, authors have used information dealing with the distribution
of human activities. The most challenging point of the process is the
estimation of the impact of each activity on each of the ecosystem
services analysed. The authors have solved this by performing a
consultation to experts. Not having empirical data that link human
activities to their impacts, authors have adopted this method for
obtaining the information that afterwards was needed to develop the
models. All the methodology is exhaustively described in the
Supplementary Material.

The methodology and the results presented could be deeply discussed and
critised, but the authors are aware of the limitations and the need of
adoption of assumptions during the implementation of the approach. For
this reason, the authors dedicate a full subsection of the paper
(Section 4.5), acknowledging the weaknesses and limitations of the
approach.

**We thank the reviewer for their thoughtful summary of our paper, and recognition of the deep uncertainties in this kind of study and our attempt at honestly reflecting this uncertainty. We also thank the reviewer for spending the time to look through our supplementary material, in which we did try to exhaustively describe our methodology.**

Specific comments
Revise and complete the affiliations.

**The primary author’s position has changed, and we have updated the affiliations according to these changes as well as to correspond with PLOS ONE’s requirements**

I miss some more information regarding to the study site to justify why
this area was selected for the research. How is been managed at present?

**We have now added an extra paragraph in the methods explaining this (lines 131-150):**

**The coast of British Columbia, Canada spans a distance of almost 1000 km, with a complex shoreline geography of fjords, inlets, and islands extending over 25,000 km in length. It is a region of diverse resource harvesting important for ecological, economic and cultural reasons, many of which are unique to the region; for example glass sponge reefs, globally significant seabird populations, salmon, eulachon, and resident orca. The region is also important culturally for intangible benefits, including nature-based tourism. A broad range of human activities occur in this region, and a multiple cumulative impact studies have been conducted to assess impacts on the marine ecosystems (8, 10, 28). Sea-based activities include fishing, aquaculture, tourism, utility and transportation. Coastal activities also influence the marine and estuarine resources in this region, including human settlement, ports and marinas, and log storage and handling. Land-based activities occurring in the watersheds are connected to coastal marine systems through freshwater runoff and include forestry, agriculture, mining and pulp and paper mills. The region is also subject to impacts from long-range and global stressors such as climate change, pollutants and debris. Activities that include vessel use additionally include the stressors associated with either small or large vessel use in their cumulative risk. Management of coastal British Columbia is siloed, with sea-based activities under the purview of Fisheries and Oceans Canada, land-based resources under provincial authority (Forest, Lands, Natural Resource Operations and Rural Development), coastal national parks under Parks Canada, and Environment and Climate Change Canada, and towns and human settlements often governed by local governments. Because of the diverse natural resources and ecosystem services, as well as the past research done on ecological impacts, we chose to study this region as a case study to study cumulative impacts on ecosystem services.**

Additional information such as the coastal length, surface or area of
the case study, depth ranges. A very short description. A couple of
lines would be enough.

**We have added some information on that in the inserted paragraph. We have added “The coast of British Columbia, Canada spans a distance of almost 1000 km, with a complex shoreline geography of fjords, inlets, and islands extending over 25,000 km in length.” (lines 131-132).**

Line 166. “Bathymetry and topography was used to calculate
viewshed”. I would say that what is below the sea surface can not be
seen and that is not part of the viewshed.

**We agree with the reviewer. We have removed reference to bathymetry, but have added that our model does include the curvature of the earth.**

Lines 272-274, would need a rephrasing to make it more clear.

**We agree with the reviewer that this sentence wasn’t clear. We have updated it to read (lines 310-315):**

**Cumulative impacts were calculated twice: first, cumulative impact scores were calculated without the service and value dimensions; next, cumulative impact scores were calculated with service and value dimensions. The difference between these two calculations reveals the contribution of considering the service and value dimensions when assessing cumulative impacts on ecosystem services.**

Section 4.5. As stated previously, this is an important section of the
paper because the authors list all the limitations and assumptions that
they had to adopt during the modelling process. Nevertheless, I still
miss one more point. The authors are assuming that the ecosystem
services production and the location of the maritime activity overlap.
This is an assumption needed when operating between different
information layers, but I think that this should be also discussed.
Certain activities could have an effect on ecosystem services that are
produced elsewhere. For example, fishing activity (e.g. bottom
trawling), could impact nursery grounds of species of commercial
interest that are fished in other locations. Thus, the impact on the
ecosystem service and the activity are not spatially coincident. The
same happens for other ecosystem services. This is a complex point of
ecosystem functioning but that it would be interesting to mention in
this section.

**The reviewer raises an interesting point about the static nature of the model and its basis in physical overlap. The current methods do address this concern somewhat, but not fully: e.g., our model incorporates an area of influence from activities based on prominent stressors from each activity. We stated so in the methods (lines 226-227), “This dataset considers the area of influence of each human activity, with the extent of each area of influence dependent on prominent stressors associated with each activity.” We also noted that the risk scoring includes a component on “community extent” which attempts at addressing some of the ecological dynamics that the reviewer raises.**

**However, we agree with the reviewer that this is still a limitation, particularly because we have represented ecosystem services (especially biologically based ecosystem services) largely from sites of human harvest. We have added the following to the discussion (lines 622-628):**

**As a simple model of cumulative impacts we also could not explicitly represent some important ecological dynamics and cascading effects that do not co-occur spatially. For example, bottom trawling can negatively affect nursery grounds for species that are fished in other locations. While we tried to capture some of these dynamics by asking experts to score the “community extent” of risk, we cannot capture the full complexity of human impact on ecosystem function, particularly where impacts affect habitats and ecosystem functions underpinning services that occur elsewhere.**

Figure 1. I think that it would be interesting to add labels to the axis
of the bar charts.

**We have now added axis labels and legends to this figure.**

Figure 2. It would be interesting to add a graphical legend indicating
what are representing the red and grey graphs.

**We have now added axis labels and legends to this figure.**

Figure 3. Add axis labels.

**We have added axis labels and a legend to this figure.**

I have added some extra comments in the Supplementary Material.

**We thank the reviewer for their thorough review. We have made modifications to the reviewer’s queries. First, we have accepted the reviewer’s changes to details in the tables. Second, regarding the scenarios used in the probabilistic inversion method, the method selects “scenarios” based on the probability distribution of ranks for exposure and consequence criteria (based on expert input), which have a vast number of possible combinations. We have added the following to the supplemental methods: “Probabilistic inversion generates scenarios by selecting from the probability distribution of exposure and consequence criteria ranks (based on expert the distribution of expert responses), which allows for thousands of nonexclusive combinations.”**

**Finally, for the reviewer’s question about the coastal vulnerability to coastal erosion, the vulnerability classes were not determined by us but are standard vulnerability data collected from GeoBC. While the reviewer asks whether a narrow sand and gravel beach should be assigned a higher vulnerability of erosion, we note that both the wider and narrow sand beaches on rock ramps have the highest erosion vulnerability scores. It is the presence of sand on rock ramps that makes for high vulnerability. The reviewer may still be right that the narrower beach should have higher vulnerability, however they both are at the highest index of vulnerability and the index does not allow for comparison within the highest index class**

Reviewer #2: The aim of the paper is very good and the subject is novel,
very interesting and very topical. Nonetheless results are presented in
a confused way: the main problem in my opinion resided in the fact taht
authors wanted to convey to much information. Some results have then
been reported in supplementary materials while several results
paragraphs deals with the same figures. As a consequence the reader is
not allowed to a full understanding.

**We thank the reviewer for their encouragement on our aims. While we acknowledge that there is a lot of information presented, we do not think it is “too much”. We thank the reviewer for their recommendation on presenting information more clearly, and we have aspired to improve this in several ways described below and above.**

I suggest to reorganise the paper and
1. to be consistent and to use the same terms in methods and results

**We have gone through the manuscript and ensured this consistency. First, we have clarified important, but related terms. We differentiate between “human activities” and “environmental stressors”, the latter as “processes that impact the environment from either human activities or long term change” (line 207). We also distinguish between risk and impact, with the former defined as “the potential of an activity to impact an ecosystem service where they co-occur” (lines 154-155).**

**We have also ensured that we are consistent in using these terms throughout the methods and results. The marked-up version of the paper will show some instances of changing terms of “drivers and activities” to “activities and stressors”, and “future impact” to “future risk”.**

2. it would be useful to have the same sections in methods and results
and in the same order of appearance

**We have gone through and ensured alignment between methods and results. Importantly, where possible we have used the same header language between the methods and results now. Note however since our methods explain the risk evaluation and mapping across multiple steps, we cannot have the same sections in the methods and results, because multiple sections of the methods will have produced a single section of results.**

3. choose most important results and information and focus on them
better explaining how they are obtained

**We thank the reviewer for their comments, but without a clear idea of what they do not understand or what they would like explained better, we do not know if we have addressed the reviewer’s concern. Regardless we have gone through the paper and ensured that we describe results. Importantly, we believe we have highlighted the most important results.**

I made comments until the results section, for discussion i would like
to see a revised verison of the paper

**We thank the reviewer for their comments, but without specific comments on the discussion we cannot be certain we are addressing their concerns. However, we have revised the paper from the introduction to the discussion and conclusion to ensure consistency and in reference to the comments of Reviewers 1 and 2.**

Here below my specific comments: please pay particular attention to
comments related to the intro section
Introduction

I appreciate that authors cite the ecosystem services cascade that
represents, in my opinion, a turning point concerning the ecosystem
services theory. Nonetheless I am not sure to share their interpretation
about the cascade.
The cascade framework was developed in order to highlight the dependence
of human well-being to ecosystems. As the authors (Haines-Young, R., &
Potschin, M. (2010). The links between biodiversity, ecosystem services
and human well-being. Ecosystem Ecology: a new synthesis, 1, 110-139.)
of the cascade say “Ecologists will increasingly have to work
alongside economists, geographers and a range of other social scientists
to understand the value that biodiversity and ecosystem services have,
to assess the costs and benefi ts of different conservation and
management strategies, and to help design the new governance systems
needed for sustainable development. Biodiversity has intrinsic value and
should be conserved in its own right. However, the utilitarian arguments
which can be made around the concept of ecosystem services and human
well-being are likely to become an increasingly central focus of future
debates about the need to preserve ‘natural capital’. The wider
research community needs to engage in such debates. Although long-term
sustainable development has come to mean many things, the concept must
include the maintenance of ecosystem services and the elements of human
well-being that depend upon healthy ecosystems. If the Ecosystem
Approach is to be embedded in decision making then we need to understand
the links between biodiversity and ecosystem services. We need to be
aware of the limits of ecological functioning and how external pressures
may impact on ecological structures and processes. Ecosystems can
exhibit non-linear responses to such pressures and the possibility of
rapid regime shifts”. It is then clear that the conservation of
natural capital intact is the basis for the maintenance of ecosystem
services at the current level. This is more and more true if we consider
that we are still not completely aware about the consequences on
ecosystem services providing (and consequently our well being) given by
the impacts imposed to the environment. In a precautionary approach and
aiming at reaching a sustainable development the natural capital should
be kept at least intact.
This is way I strongly disagree with certain assumption of authors, for
instance when they state:
“ Impacts to ecosystem services are potentially different than impacts
to ecosystems. While impacts to ecosystems have (usually adverse)
consequences for populations,species, and structure of ecosystems,
impacts to ecosystem services ultimately negatively affecthuman
wellbeing. For exa mple, pollution might not affect shellfish growth,
but it may lead to aquaculture tenure closure for health concerns, or
might affect the taste of shellfish caught at polluted sites (14).
Changes to the enjoyment of shellfish aquaculture, in this case, are not
a result of changes in the biophysical supply of the service but in the
change to either the access to or quality of the ecosystem service (i.e.
environmental impact may not mean ecosystem service” “Reframing the
previous example, pollution impacts the service (the ability of people
to access shellfish for food through legal restriction) or the value
(the palatability of the shellfish), but the growth of shellfish (the
supply) is unaffected.”
On the contrary I agree about the concept that studying or mapping
ecosystem services fruition is strongly different from studying and
mapping ecosystem services supply (that in the cascade coincide with
ecosystem functions that represent the capability of ecosystems to
provide services).
If authors want to focus on the “human side” of the cascade it is ok
and in my opinion an interesting issue given that a great confusion
still exist in how represent and map ecosystem services themselves.
Often researches represent ecosystem services confusing them with
ecosystem functions and then it is important to give tools to represent
services. In my opinion the introduction should been rewritten focusing
on this latter aspect rather than trying to demonstrate that ecosystem
services’ supply can not be affected by modifications to natural
environment.
At this purpose I suggest the reading of Burkhard B, Maes J (Eds.)
(2017) Mapping Ecosystem Services. Pensoft Publishers, Sofia, 374 pp.

**We thank the reviewer for this thoughtful feedback. We completely agree with the reviewer that ecosystem services are modified by the natural environment. We realize that stating that “impacts to ecosystem services are potentially different than impacts
to ecosystems” can be read as saying that impacts on ecosystems are not impacts on ecosystem services, and so we have removed that statement and clarified our point. We did not mean to suggest that environmental change does not affect ecosystem services, but rather that in order to understand impacts to ecosystem services, we need to think beyond only thinking about environmental changes. We emphasize this when we write: “While any human activity that impacts ecosystems has the potential to impact ecosystem services, ecosystem services includes dynamics beyond the ecological production of potential benefits to people” (lines 61-63). However, we can see how the previous text was unclear on this point and have modified text in the introduction to clarify our intended meaning. On a further point, while we do recognize the work of Haines-Young, R., & Potschin, M. (2010) on the concept of ecosystem service cascades, we rather operationalize a more recent version of the cascade framework, by Tallis et al. (2012). This more recent framing is explicitly focused on the interplay between social and biophysical dimensions of ecosystem services, as is the focus of our work. Finally, note that—contrary to the reviewer’s comment—our work extends well beyond the “human side” of the cascade, since much of our modeling work involves both the biophysical basis of ecosystem services. We have changed text in the introduction to read as follows (lines 63-73):**

**Assessing impacts to ecosystem services therefore must include considerations beyond changes to the natural environment. The dynamic interactions between environmental change and human beneficiaries of ecosystem services may in some cases mean that ecosystem services are degraded without substantial impacts on the underlying environment, and in other cases that the underlying environment is degraded without substantial impact on ecosystem services. Differences in how ecosystems and ecosystem services are impacted is largely unexplored in the literature, however. For example, pollution might minimally affect shellfish growth, but it might lead to aquaculture tenure closure for health concerns, or affect the taste of shellfish caught at polluted sites (****14****). Changes to ecosystems’ contribution to shellfish aquaculture, in this case, are not a result of changes in the biophysical supply of the service. Rather, the ecosystem service is impacted via either the access to or quality of the service.**

Methods
I suggest to list the considered ecosystem services always in the same
order: these makes easier to the reader to keep them in mind

**We have gone through the manuscript and made sure that lists of ecosystem services are presented in the same order.**

Lines 133-141 please insert a numbering list

**We have now made this a numbered list**

Line 151: please briefly introduce somewhere in the text the invest
model

**We have now added the following text (lines 180-183): “InVEST (integrated valuation of ecosystem services and tradeoffs) is a decision-support tool for mapping and valuing ecosystem services, that generates spatially explicit models of ecosystem services based on underlying ecosystem characteristics.”**

line 154: authors should better specify what marine recreation is for
instance listing all the activities composing the services as done in
Supplementary materials

**We have now added the following text (line 186-187): “Marine recreation includes kayak, recreational boating, recreational fishing, and populous sites for recreation, including camping and dive sites.”**

Line 174: should specify what to they mean with the word “stressor”
and I suggest to choose only a name (activity or stressor or a third new
word)

**We specifically differentiate activities and stressors because we include climate change risk, and erosion, which are processes not associated with a single human activity. We have chosen to define the term as “processes that impact the environment from either human activities or long term change” (line 207).**

Paragraph 2.2 should be rewritten since it is unclear.

**We have tried clarifying the section but we cannot be certain it satisfies the reviewer (changes can be viewed in the marked-up version of the manuscript). We would be happy to make additional revisions based on further guidance.**

Table S2 should
be modified and moved to the main text: here are listed the ecosystem
services and the stressor but the list of ecoservices is useless since
the readier already knows it while it is not highlited the relationships
between ecoservices and stressors. It would be more useful to list
stressors in the first column and match each stressor with the affected
ecoservices. It should be explained which indicator was used to
calculate the intensity of each stressor.

**We have moved the table to the main text. We agree with the reviewer on the change to the structure and have done so here. However, given the complexity of the table with including the indicator (all ecosystem services have multiple activities and stressors associated), it would be too difficult to attach indicators. We have left this information for the supplement**

Line 183-185: unclear, rephrase

**We have rephrased the sentence to read, “We treat ecosystem services as broad categories (such as demersal vs pelagic fisheries) and activities and stressors that cause impact as specific categories because experts indicated that broad types of ecosystem services (such as various benthic fisheries, or various pelagic fisheries) are impacted in similar ways, while they indicated that they did not treat human activities and stressors in a similar way. ” (lines 215-219).**

Line 190-192: I should remove this sentence, as it is it seems to be
useless

**We disagree with the reviewer here but would be interested to better understand the reviewer’s thinking. In our opinion, the finding by Ban et al (2010) is useful methodologically, and explains why we chose to treat our data the way we did. As we write, “the number of data layers influences the overall cumulative impact scores (****8****), and we did not want to overly bias impact based on fisheries scores” (lines 224-226).**

Line 264-267: this sentence is unclear, may be a formula would help?

**We have clarified this sentence to read, “All intensity data for human activities were log transformed and normalized by dividing by the largest intensity value found for each activity and stressor across the BC coast to generate a dimensionless 0-1 intensity scale” (lines 302-304).**

Results
Results in Figure 1 and 2 are very interesting but if intensity scores
are plotted on the instogram it should be explained in the figure or in
the caption moreover it should be more interesting to set the upper
limit of the color scale to the same value in order to allow the reader
to compare the maps.

**We have added more description to the plots (something that the first reviewer also mentioned) and have ensured that the caption also describes that the figure shows the maps and the total impact. Note that some ecosystem services register total impact values an order of magnitude higher than other ecosystem services, and so putting all graphs on the same upper limit would make some graphs very hard to read.**

In this case a table with the higher cumulative impact reached by each
ecosystem service could be added.

**We are not sure what the reviewer means by this statement. Does the reviewer suggest adding a table ranking the activities and stressors for each ecosystem service by how much impact they contribute? We feel that would be largely redundant with figures 1 and 2, and the text in the results, which already showcase the cumulative impact scores by activity and stressor, and provides cumulative impact scores.**

Lines 292-295: please add a table with these scores

**We have considered it and decided that adding another table would be redundant with figure 3 (a redundancy that the journal has stated we should avoid), so we have retained the ranked list.**

Figure 3 must be better explained, it is not clear what it is plotted
and its meaning. I suggest to authors to be consistent and use in the
results exactly the same terminology explained in methods. If some
metrics presented in results are not explained in methods authors must
add an explanation.

**We have recreated the figure with more explanation of the axes, something reviewer 1 also mentioned.**

Lines 323-327: please add table where the density of IC (see previous
comment- Lines 292-295: please add a table with these scores-) and total
Ic are reported

**As noted previously, we believe these tables would be largely redundant with information already presented in figures. We have decided not to add this table.**

Line 327-336: not clear please rephrase: where can I see it? Not clear
were this ranking is introduced. May be is this reported in appendix? In
case it should be moved to results or removed at all.

**The ranking is discussed in the text. We have added the word “ranked” at the beginning of this section so it is further clarified. We have clearly stated where we rank for both total impact scores and per-cell impact.**

Figure 4 is useless please replace it with a table.

**While we respect the reviewer`s preference for a table, we disagree that figure 4 is “useless” as it clearly showcases trends. We have workshopped how to represent this data and found this type of graph best for showcasing the results.**

Paragraphs 3.3 and 3.4 page 16: the numbering of paragraph is wrong:
these are 3.5 and 3.6 paragraph

**We thank the reviewer for pointing this out. We have fixed the numbering**

-------------------------
