# Supplementary material for: Mapping cumulative impacts to coastal ecosystem services in British Columbia
Source: PLoS One. 2020 May 4;15(5):e0220092. doi: 10.1371/journal.pone.0220092 (PMC7197858; doi:10.1371/journal.pone.0220092)
Supplement: Attachment — Submitted filename: responses_to_reviewer_PLOSONE_v2.docx [file pone.0220092.s012.docx]

**We thank the editor and the reviewer for their feedback and willingness to clarify points raised. Without this further clarification we believe we would not have been able to properly address the reviewer concerns. We have taken this opportunity to clarify our manuscript throughout and ensure we are consistent with our terms and definitions. Below you will find our responses to the particular reviewer comments in bold red**

Reviewer #2: I did not find the modification made by authors completely effective: most part of my comment have been ignored, hopefully since authors did not completely understand them. The paper, to me, cannot be published as it is and still need a strong revision and several modifications to be published. Here below my new comments, I tried to be clearer. I suggest the authors to put a diagram of their theoretical path in methods to clarify: aims, methods, results and re-arrange the paper accordingly.

**As we have been in contact with the reviewer following this review, it became clear that there were misunderstandings on both sides. We thought that we had responded to all of the reviewer’s concerns in the last round, but the reviewer thought that we ignored them. It has become apparent to us that we did not understand the reviewer’s comments in full, and so our responses did not fully address the reviewer’s comments. We have now further clarified our framing of ES theory and removed sections that might cause misunderstandings. There may still be some disagreement over ES theory, so we provide extra examples and literature to back up our points in this response.**

**We agree with the reviewer that having a diagram to give a high-level approach to our study is useful. We have now included such a diagram (Fig 1).**

Introduction
I made a long comment to explain the authors that some sentences about the interactions among services and environment should have been changed buit they ignored it. As a consequence I strongl suggest to remove lines from 68 to 78 “Assessing….service”(version of the paper with track changes). Analogously lines from 90 to 98 (“Reframing...an impact to an ecosystem service”) must be removed: they are disorganized and misleading. I suggest the authors to find in literature some theoretical example of the information they want to convey. Please put references when reporting this. Modifications made do not solve the issue.

**Based on further exchanges with the reviewer after receiving this feedback, we think we now understand the reviewer’s perspective and have addressed it. Our primary point here was that impacts to ecosystem services can be social in nature (via access and demand/perceived quality), which may add impact on top of biophysical changes. Theoretically, there can also be cases where changes to people’s access and demand/perceived quality of ecosystem services impacts ecosystem services without undermining the biophysical provision. We have clarified with the reviewer via email that his/her primary concern was that it is important to be precautionary and not suggest that changes to ecosystem services do not imply changes to the underlying ecosystem. We do not wish to convey that impacts to the underlying ecosystem do not impact ecosystems services (we think they usually do, and they always affect the potential of future ecosystem service enjoyment), and so we have simplified and clarified the text here.**

**For further clarification of our perspective (only if this is needed), here’s more detail with examples. While ecosystem service supply is always important because you cannot have ecosystem services without their supply, this does not mean that all impacts to ecosystem service as benefits are mediated by supply. In perhaps the most famous example of ecosystem service change being driven by changes in access and preferences (and not the ecosystem), pollinating services for coffee in Costa Rica first decreased as coffee prices dropped then were rendered worthless as farmers switched from coffee to pineapple (a more valuable crop which is wind pollinated and not animal pollinated). The potential for pollination services was still there (if in the future animal pollinated crops were planted again), but because of economic choices the value of pollinators decreased (McCauley 2006). Other examples of ecosystem services changed by human access and value exist, and some are provided right in the literature we refer to (e.g. Tallis et al. 2012). Examples from Tallis et al. 2012 include that shoreline protection from kelp and the provision of wave energy as ecosystem services are regulated by the existence and location of human built infrastructure. Changes to the location and existence of this infrastructure will change the ecosystem services being delivered to people without necessarily affecting the underlying ecosystem that is providing the potential service.**

**The reviewer has also strongly suggested that we refer to a theoretical example, and so to avoid more disagreements about the example we previously provided about shellfish production, we have instead relied on examples that we document in other peer reviewed publications (Singh et al. 2017). Our text now reads (lines 64-75):**

**Any human activity that impacts ecosystems has the potential to impact ecosystem services in multiple ways. In addition to impacts on the biophysical production of services, human activities and infrastructure can also undermine the “consumption” of ecosystem services [****14****].That is, a human activity can undermine people’s ability to access or enjoy an ecosystem service. The role of impacts to the production versus the consumption of ecosystem services is largely unexplored in the literature, however. For example, in New Zealand shellfish aquaculture sites and shipping lanes can limit commercial fishing operations in an area because of legislation that limits their overlap, impacting the contribution of fisheries ecosystem services [****15****]. In this case, the assessed impact of shipping and aquaculture on fisheries operated through changes in access and not through impacts on biophysical supply (though the effluent from increased shipping may impact biophysical supply in the long term).**

**We have also changed the text revisiting the example in the framework of ecosystem service supply, service, and value, to read (lines 83-93):**

**Impacts to ecosystem services can be characterized at each step in the ecosystem services ‘cascade’ [****13****], with impact drivers potentially affecting supply (the biophysical components that produce ecosystem services), service (the ability of people to access and benefit from a service), and value (people’s preferences for ecosystem services, 13). Reframing the previous example, shipping lanes and aquaculture sites impact the service (the ability of people to access fisheries for food through legal restriction), even if the growth and availability of fish (the supply) might be unaffected. In this case (where shipping lanes and aquaculture restrict fisheries) what might not be considered an environmental impact (to fish) would be considered an ecosystem service impact. While this cascade is useful for parsing out the dynamics of impacts to ecosystem services, the relative importance of these factors (supply, service, and value) in regulating impact to ecosystem services is not known.**

**McCauley DJ (2006) Selling out on nature. Nature 443: 27-28.**

**Singh GG, Sinner J, Ellis J, Kandlikar M, Halpern BS, et al. (2017) Mechanisms and risk of cumulative impacts to coastal ecosystem services: An expert elicitation approach. Journal of environmental management 199: 229-241.**

**Tallis H, Lester SE, Ruckelshaus M, Plummer M, McLeod K, et al. (2012) New metrics for managing and sustaining the ocean's bounty. Marine Policy 36: 303-306.**

I also suggested to cite Burkhard B, Maes J (Eds.)
(2017) Mapping Ecosystem Services. Pensoft Publishers, Sofia, 374 pp. But it has not been done

**We have now added this citation**

They say that their approach is based on Tallis et al. 2012 (moreover they theory they are referring to is better explained, in my opinion in Tallis et al. 2012, “A Global System for Monitoring Ecosystem Service Change”, rather than in “New metrics for managing and sustaining the ocean's bounty”) but they discuss Heines-Young. As a consequence I strongly suggest to better refer to Tallis et al. (2012) and to deepen their theory without referring to the cascade.

**As we have noted in our previous contact with the reviewer, the reviewer is suggesting that we cite a different paper, one that actually cites the paper that we cite *as the primary and most comprehensive source of the theory*. Accordingly, we have retained our references to Tallis et al (2012) “new metrics for sustaining the ocean’s bounty”. We have also added the citation to the paper the reviewer suggests, however, because it is a paper based on monitoring ES change, which is also relevant to our paper. Our responses above address our attempts to further address the theory put forward by the literature. Finally, given the further exchanges with the reviewer, we believe that both sides (us and the reviewer) see the ecosystem cascade theory as well as that proposed by Tallis et al (2012) to be largely complementary.**

The Introduction must then be rewritten, as I already suggested.

**The introduction has been revisited and rewritten again now to address the points brought up by the reviewer.**

Lines 122-128: unclear, please rephrase

**We agree that these lines were not as clear as they could have been. I have updated the following lines for clarity, focusing on how linking the ideas of ecosystem service supply, service, and value dimensions requires a better causal understanding of impacts. The text now reads (lines 114-117):**

**However, changes to people’s access, use, and perceived quality of service may also be important for understanding impacts to ecosystem services [****27****,****28****], and understanding the mechanism of impacts on ecosystem services can help address management goals [****17****].**

Lines 132-133: the authors say that their aim is to know Which ecosystem services face the greatest cumulative impact in coastal British Columbia but the reader still don’t know what they mean with cumulative, what does it means that “the ecosystem services face the greatest”
The underlying theory must be explained more clearly

**We agree that we can better describe what “cumulative” impacts are. We have now added an explicit definition for cumulative impacts in the opening paragraph (lines 50-53):**

**The need to understand and manage simultaneous impacts of multiple human activities on ecosystems (such as fisheries and agricultural runoff impacting fish habitat concurrently), referred to here as cumulative impacts, has led to widespread uptake in cumulative impact mapping methods around the world [****3****,****4****,****5****,****6****,****7****,****8****,****9****,****10****,****11****].**

**We also explicitly state how we define cumulative impacts with regards to our study (lines 124-127): “****This work, alternately, does so for ecosystem services themselves, representing cumulative impact as the combined total impact that an ecosystem service experiences from a variety of co-occurring drivers of impact (such as ocean acidification, agricultural runoff, and fishing)”. This framing largely adapts the theory from Halpern et al (2008, 2015).**

**We have also changed the language away from “the greatest” impact to “the most severe impact”, which we think clarifies the concept, especially in the context of our definition of the combined total impact that ecosystem services experience.**

**Halpern BS, Walbridge S, Selkoe KA, Kappel CV, Micheli F, et al. (2008) A Global Map of Human Impact on Marine Ecosystems. Science 319: 948-952.**

**Halpern BS, Frazier M, Potapenko J, Casey KS, Koenig K, et al. (2015) Spatial and temporal changes in cumulative human impacts on the world’s ocean. Nature communications 6: 1-7.**

Line 157: not clear to me what the authors mean with “siloed”

**By “siloed” we refer to the notion that managing different ecosystems and industries is done in a piecemeal way, so that, for example fisheries and agriculture are managed independently with little to no coordination between regulating bodies. We have changed this text to replace “siloed” with “in a piecemeal way”**

Methods

I suggested to reorganise and be consistent. Authors list 5 phases: since subsequently 5 subparagraphs are present I strongly suggest to name each paragraph accordingly to each phase listed
e.g. if the paragraph 2.3 is “spatial representation of ecosystem services” in the list at line 172 it should be written: “spatial representation of ecosystem services: we mapped eight ecosystem services…..”

**We have now added the header to begin each line that introduces each phase, as suggested by the reviewer**

Line 189: it is not clear which variables have been mapped, please inserted a table with very brief description for each service or insert an explanation in the text

**We have a supplemental table with all the variables used. We have reorganized this table so that the variables are associated with each relevant ecosystem service (S2 Table). We have referred to this table in the text and we have added brief description of each ecosystem service in the text (lines 204-214):**

**Coastal aesthetics was modeled by calculating the viewshed from kayaking, recreational boating, population centers, recreational fishing. Coastal protection was modeled assessing the protection provided by marine vegetation (kelp and seagrass) to different types of shoreline (sandy to rocky). Benefits from commercial demersal fisheries and benefits from commercial pelagic fisheries were modeled by aggregating multiple commercial fishery spatial data layers. Coastal recreation includes kayak, recreational boating, recreational fishing, and populous sites for recreation, including camping and dive sites. We modeled “potential” energy generation because British Columbia currently does not have wave and tidal energy operations, but there is interest in harnessing this energy supply. Benefits from finfish and shellfish aquaculture were modeled by aggregating spatial data of finfish and shellfish aquaculture. For more detail on the ecosystem service models, see Supporting Methods.**

Lines 191: please list the services here so move here lines from 195 to 198.

**We have moved these lines up to line 191 (now lines 197-201)**

If I correctly understand only renewable energy and aquaculture were modelled without using INVEST. It can be easily said, at lines 191-192: the eight services were all modelled using InVest excluding energy and aquaculture for which we used the publicly available spatial.

**We have now stated the above, as the reviewer mentions**

Still not clear to me the difference between services and human activities or stressor (please choose a unique term).

**Services refer to ecosystem services, while human activities or stressors refer to the causes of impact. We use these terms because they are widely used in the cumulative impacts literature (Murray et al 2016, Singh et al, 2017). We have changed the term to “drivers” to capture the activities and stressors (also a term used in cumulative impacts literature) and have made sure we use the term consistently throughout.**

**We have expanded on our definitions of ecosystem services, drivers, and impacts. We have added the following definitions:**

**“Ecosystem services are the environmental processes that render benefits to people. Implicit to this definition is that, while ecosystem functions are essential for providing ecosystem services, these services do not exist without human beneficiaries [****13****,****14****].” (lines 62-64).**

**“****We define drivers as the human activities and long-term stressors (such as ocean acidification) that contribute to a deterioration of benefits derived from ecosystem services. We define stressors as the processes that undermine ecosystem service benefits, and we define impacts as the deterioration of ecosystem service benefit. For example, agriculture contributes to runoff that can lead to sedimentation which can smother shellfish harvested by people [****15****,****16****]. In this example, agriculture is a driver, sedimentation is a stressor, and reduced shellfish biomass for food is the impact [****15****,****17****].” (lines 77-83)**

**Murray CC, Mach ME, Martone RG, Singh GG, O M, et al. (2016) Supporting risk assessment: accounting for indirect risk to ecosystem components. PLoS ONE 11: e0162932.**

**Singh GG, Sinner J, Ellis J, Kandlikar M, Halpern BS, et al. (2017) Mechanisms and risk of cumulative impacts to coastal ecosystem services: An expert elicitation approach. Journal of environmental management 199: 229-241.**

I think these definition should be given with a very clear example of each definition
So please define and give an example of:
- ecosystem services
- impacting activities
- Risk

I know they state they defined these in the text but definitions are sparse and not clear.

**We have clarified these definitions in the text. See the above definition for definitions of ecosystem services, drivers (formerly impacting activities) and impacts. We have always had a definition of risk in the manuscript, which we state is “the potential of a driver to impact an ecosystem service where they co-occur”. We have also further clarified our definition of risk, adding the following to the section describing the cumulative impacts model: “****We define risk as the potential of a driver to impact a particular ecosystem service. In the context of our cumulative impact model, risk is the potential of a single event of an activity to impact a given ecosystem service.” (lines 266-268).**

Moreover it is confusing to me that fisheries are both a service and a stressor, this should be clarified.

**We have clarified this by explicitly pointing out that fisheries can be treated as both ecosystem services and stressors, because fisheries harvest fish biomass for food, but fisheries also damage underlying ecosystems and can reduce access and quality of ecosystem services. The existence of fisheries as both ecosystem service and stressor/impacting activity is established in the literature (Raymond et al. 2013, Singh et al. 2017). When we refer to fisheries as ecosystem services we are actually recognizing the “benefits from fisheries” as the benefits that humans derive from ecosystems through fisheries harvest. When we refer to fisheries as stressor, we are recognizing the impacts to ecosystem services that fisheries generate. We have referred to “benefits from fisheries” throughout the text now, to help clarify the difference. We have added the following text (line 237-244):**

**Many human activities, such as fishing, access benefits from ecological processes and play important roles in ecosystem service delivery to people while also contributing impacts towards ecosystem services [****15****]. We treat these activities (e.g. fishing), therefore as both ecosystem services as well as drivers that cause impact (following Singh et al. [****15****]). To distinguish between these multiple roles that fisheries play, we emphasize benefits when labeling fisheries as ecosystem services (such as “benefits from commercial demersal fisheries”) and emphasize impacts when labeling fisheries as drivers of impact (such as “demersal destructive fishing”).**

**Raymond CM, Singh GG, Benessaiah K, Bernhardt JR, Levine J, et al. (2013) Ecosystem services and beyond: Using multiple metaphors to understand human–environment relationships. BioScience 63: 536-546.**

**Singh GG, Sinner J, Ellis J, Kandlikar M, Halpern BS, et al. (2017) Mechanisms and risk of cumulative impacts to coastal ecosystem services: An expert elicitation approach. Journal of environmental management 199: 229-241.**

Lines 250-253: this should be in some way explained before when the concept of cumulative impact is introduced.

**Since we have now defined cumulative impacts, we hope this helps clarify this point. We have also added the following to the methodological overview (lines 187-190):**

**we overlaid maps of drivers of impact (with impact scores) on maps of ecosystem service to assess the cumulative impacts of all available activities on each service, in accordance with our definition of cumulative impacts.**

Results
In my opinion results should be simplified and better presented
First, authors should avoid to list indicator values in text as list, please use table. Moreover presenting impact scores were calculated before without the service and value dimensions and later with service and value dimensions makes difficult to read the paper. I suggest to remove this or to present it in clearer way, for example with two separate sessions. Moreover as I already told authors should better explain in method what are these “dimensions”: supply dimensions, values dimensions...there are too many concepts and terms and this confusing. As I already suggest in the first review is necessary to focus on some main results they want to convey and present them in a clearer and synthetic way.

**As clarified by the reviewer on further communication, we have removed the in-text results and instead pointed to the relevant figures which showcase the results. We have also added a table which shows all the impact scores and refer to this table (Table 2). We have also further explained the supply, service, and value dimensions in the introduction as demonstrated in an earlier comment. Finally, we have separated results of ES supply service and value and results of just ES supply by incorporating another header level.**

Moreover I think that the part regarding InVEST, mapping and the cumulative index should be clearly separated in both methods and results and merged when put together to make the cumulative impact. I think this should be a good framework for the entire paper:
-maps
- expert
-cumulative measures

**We have considered this suggestion but cannot fully implement it because our results do not separate the InVEST mapping and the cumulative impact scores. The cumulative impact mapping framework that we adopt here (from Halpern et al 2008, 2009, 2015) does not separate the maps, expert values and cumulative impacts but synthesizes them all to produce maps of impact.**

**However, we have ensured that our methods are largely put together in the sequence that the reviewer suggests. We agree with the reviewer that this structure is useful for the methods. We have moved the methods sections on future risk and impact mechanisms to follow the expert elicitation for risk scores because these are also expert derived.**

**Halpern BS, Walbridge S, Selkoe KA, Kappel CV, Micheli F, et al. (2008) A Global Map of Human Impact on Marine Ecosystems. Science 319: 948-952.**

**Halpern BS, Kappel CV, Selkoe KA, Micheli F, Ebert CM, et al. (2009) Mapping cumulative human impacts to California Current marine ecosystems. Conservation letters 2: 138-148.**

**Halpern BS, Frazier M, Potapenko J, Casey KS, Koenig K, et al. (2015) Spatial and temporal changes in cumulative human impacts on the world’s ocean. Nature communications 6: 1-7.**

Lines 350-355 should be put in a table

**We have pointed to the relevant figure, as well as the new Table 2 which shows these values.**

Line 355: is the supply dimension identified by identified by spatial representation of ecosystem services? Please be consistent: it must be clear to which part of the methods the authors refer in results

**Since we have further clarified what ecosystem service supply, service, and value dimensions are in the introduction and methods, we believe we have clarified this further. The supply dimension is looking at impact only considering impact to the biophysical system that produces the ecosystem service and not the service and value dimensions that consume the service. We have also further clarified this line to read (line 385-388): “When mapping the per-cell cumulative impact model while only considering impacts to ecosystem service supply (and not including service and value dimensions), the ranked list of ecosystem services facing impacts is similar to the list considering service and value dimensions, with some differences.”**

Lines 357-381: need table with Ic

**We have pointed to the relevant figure and table for the Ic values in text. Note that lines 362-381 (now 394-417) are figure captions and so implicitly refer to the figures.**

Line 382: how is it calculated the “total summed impact”, insert also these values in a table. It is not necessary to list all values in the text and this makes the reading very hard. If values are diffent for only supply dimension and not please insert both

**We understand this term can be confusing. To be more consistent, we have changed this to “cumulative impact” and refer to the calculation of *I_c_*. We have also removed the in-text results and pointed to the relevant figures (and Table 2) which show these data.**

Lines 391:where can I see this? Fig. 1? If yes please cite here

**We have cited the figures now**

Lines 421-430: again, these results should be presented with a table, directly compared with the previous ones. Please name the different Ic with different names: the reader reads all along the text Ic but if I correctly understand this sometimes refers to the index with value dimension, sometimes not.

**We have removed the in-text results here and put them in a table for both the per-pixel and total Ic values (Ic across spatial range), since no figure clearly shows the proportional change in the impact scores when considering ecosystem service service and value dimensions on top of supply dimensions. We have also labeled the per-pixel Ic values and the total Ic values across the spatial range to differentiate them. We have also ensured that when we refer to Ic values that include service and value dimensions we indicate this, and when we refer to Ic values that do not include service and value dimensions we refer to Ic values for supply dimensions.**

Figure 6: it should be better explained in methods how these values are obtained

**We have rewritten the section in the methods to describe how we gathered this data from experts. The section now reads (lines 340-352):**

**We asked experts in the risk survey to indicate whether or not the given drivers of impact affected their chosen ecosystem service directly or indirectly (or neither or both), with an optional follow-up to describe the mechanism of impact. Each driver of impact were grouped in one of four different categories: fisheries impacts, coastal commercial impacts, land-based impacts, and climate change impacts. Fisheries impacts includes all those drivers related to fisheries including demersal destructive and non-destructive fishing, pelagic fishing, and recreational fishing. Coastal commercial impacts include coastal industries such as aquaculture, shipping, ports, docks, log dumping, ocean dumping. Land-based impacts include industry, pulp and paper, onshore mining, human settlements, forestry, and agriculture. Climate change impacts include ocean acidification, sea level rise, sea temperature change, and UV change. When all drivers were categorized, we calculated the proportion of direct versus indirect impacts (also accounting for impacts that could be both or neither) within each category affect each ecosystem service.**
